# Three‐dimensional structures of avian beta‐microseminoproteins: insight from the chicken egg‐specific beta‐microseminoprotein 3 paralog

**DOI:** 10.1002/2211-5463.13166

**Published:** 2021-05-24

**Authors:** Franck Coste, Thierry Moreau, Valérie Labas, Magali Chessé, Mégane Bregeon, Hervé Meudal, Karine Loth, Bertrand Castaing, Nicolas Guyot, Sophie Réhault‐Godbert

**Affiliations:** ^1^ CBM CNRS UPR4301 Orléans France; ^2^ INRAE Université de Tours BOA Nouzilly France; ^3^ INRAE CNRS IFCE Université de Tours PRC Nouzilly France; ^4^ INRAE CHU de Tours Université de Tours PIXANIM Nouzilly France; ^5^ UFR CoST Université d’Orléans Orléans France

**Keywords:** beta‐microseminoproteins, birds, crystal structure, egg, MSMB3, paralogs

## Abstract

Beta‐microseminoproteins (MSMBs) are small disulfide‐rich proteins that are conserved among vertebrates. These proteins exhibit diverse biological activities and were mainly reported to play a role in male fertility, immunity, and embryogenesis. In this work, we focused on the chicken MSMB3 protein that was previously depicted as an egg antibacterial protein. We report that MSMB3 protein is exclusively expressed in the reproductive tissues of laying hens (in contrast to chicken MSMB1 and MSMB2 paralogs), to be incorporated in the egg white during the process of egg formation. We also showed that chicken MSMB3 possesses highly conserved orthologs in bird species, including Neognathae and Palaeognathae. Chicken MSMB3 was purified from egg white using heparin affinity chromatography and was analyzed by top‐down and bottom‐up proteomics. Several proteoforms could be characterized, and a homodimer was further evidenced by NMR spectroscopy. The X‐ray structure of chicken MSMB3 was solved for the first time, revealing that this protein adopts a novel dimeric arrangement. The highly cationic MSMB3 protein exhibits a distinct electrostatic distribution compared with chicken MSMB1 and MSMB2 structural models, and with published mammalian MSMB structures. The specific incorporation of MSMB3 paralog in the egg, and its phylogenetic conservation in birds together with its peculiar homodimer arrangement and physicochemical properties, suggests that the MSMB3 protein has evolved to play a critical role during the embryonic development of avian species. These new data are likely to stimulate research to elucidate the structure/function relationships of MSMB paralogs and orthologs in the animal kingdom.

## Graphical abstract

Beta‐microseminoproteins (MSMBs) are small proteins conserved among vertebrates. The chicken genome contains three MSMB paralogs, which exhibit distinct tissue expression profiles, with chicken beta‐MSMB 3 (MSMB3) protein being egg‐specific. Collectively, results from protein sequence analysis, mass spectrometry analysis, and 3D structure data highlight that chicken MSMB3 possesses specific features that may be related to its presumed role in avian reproduction.

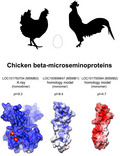

**Abbreviations**
MALDI‐TOF: matrix‐assisted laser desorption ionization–time of flight
MSMB: microseminoprotein
nanoLC‐MS/MS: nanoflow liquid chromatography–tandem mass spectrometry
PDB: protein data bank
PSP94: prostate secretory protein 94
SSP‐2: small serum protein 2
TOCSY: total correlation spectroscopy

Beta‐microseminoproteins (MSMBs) are nonglycosylated disulfide‐rich miniproteins (molecular mass about 10 kDa) that have been identified in many animal species [1, 2]. MSMB genes have been reported to exhibit strong conservation among vertebrates, although they share a relatively low level of sequence identity [1, 3, 4, 5]. This observation supports that MSMB ancestor gene has likely undergone a complex pattern of evolution consisting of sequence changes, amino acid substitutions, and duplication events [1, 6, 7]. All ten cysteine residues of MSMB are conserved and engaged in specific disulfide pairings [8]. Moreover, the C‐terminal domain contains two two‐stranded β‐sheets with no resemblance to known structural motifs [9]. Only tridimensional structures of mammalian (human, porcine, and bovine species) or snake MSMBs have been solved [9, 10, 11, 12] to date.

The human prostate secretory protein 94 (PSP94, also named beta‐MSMB, or beta‐inhibin) is considered as the archetype of MSMB proteins. Its amino acid sequence has been published in 1985 [13]. The human PSP94 is a sperm‐coating antigen isolated from human seminal plasma, and its high expression in the prostate gland [14] has motivated many researchers to evaluate its relevance as a biomarker of prostate cancer [15]. Regardless of the species, MSMB proteins have been essentially identified in mucous glands and secretions [16]. Their known biological functions are essentially associated with male fertility and include spermatozoon maturation/capacitation (by binding to cysteine‐rich secretory protein, CRISP3) and acrosome reaction [17, 18, 19, 20, 21, 22, 23, 24]. Only one article reports a role in female reproduction [25]. Besides these physiological functions, some MSMB proteins are assumed to participate in innate immunity, considering their activity against *Candida* pathogenic yeasts and bacteria [26, 27], while other MSMB proteins were reported to display lymphocyte‐stimulating activities [28, 29]. In parallel, some members of this protein family bear antitoxin properties, through the binding to secretory toxins that are present in snake venoms [10, 21]. In avian species, a MSMB protein has been identified in the pituitary gland of ostrich, but its physiological function has not been characterized yet [7]. Three chicken paralogs named MSMB1, MSMB2, and MSMB3 localized on chromosome 6 and flanked by WASHC2C (alias FAM21C) and NPY4R (alias PPYR1) genes have been described previously [1]. The function and the tissue distribution of chicken MSMB1 (LOC101750594) are not known. In contrast, chicken MSMB2 (LOC100858647) has been identified in the eggshell [30] and in both sperm and seminal plasma of male chickens [31]. The localization of chicken MSMB2 in male semen is consistent with a potential role of chicken MSMB2 in male fertility, similarly to mammalian MSMBs. Chicken MSMB3 (LOC101750704) was first purified from egg white and was reported to exhibit antibacterial activity against *Listeria monocytogenes* and *Salmonella enterica* Enteritidis [26, 32]. To our knowledge, chicken MSMB1 and MSMB2 have never been identified in egg white, nor in egg yolk. From these scarce data in avian species, the functions of chicken MSMBs in male reproduction and immunity resemble those described for mammalian MSMBs. Interestingly, some published articles underlined a potential role of chicken MSMB proteins in the early stages of chicken embryonic development, specifically during the formation of mesodermal structures [33]. In addition, a homolog of chicken MSMB2 that was characterized in amphioxus (29% protein sequence identity) was reported to be potentially involved in the differentiation of ectoderm during embryonic development [34], and likewise, in Xenopus, a MSMB protein was shown to be essential to regulate neural crest migration [35]. The high variability in MSMB protein sequences that has arisen during speciation is likely associated with distinct physicochemical properties and potentially distinct tridimensional structures, which may ultimately result in diverse biological activities. As an example, the heparin‐binding domain of chicken MSMB3 seems to be involved in the antibacterial activity of the protein [26].

In the present article, we focused on the three chicken MSMB paralogs. We first evaluated the tissue specificity of the three paralogs in male and female chicken tissues. We also compared the chicken MSMB1, MSMB2, and MSMB3 protein sequences and searched for MSMB3 orthologs in other avian species. We showed that MSMB3 protein sequence is highly conserved in bird species, in contrast to the other MSMB proteins that are present in many vertebrates. MSMB3 purified from chicken egg white has been analyzed by mass spectrometry to verify its protein sequence and identify proteoforms, and by NMR to assess its behavior in solution. The X‐ray structure of MSMB3 has been solved and compared to (a) published structures and (b) chicken MSMB1 and MSMB2 model structures built by homology modeling. Altogether, our data highlight some MSMB3‐specific features, which suggest that this protein plays a crucial role in avian reproduction, although its precise function in the egg remains puzzling.

## Results

### The chicken genome contains three MSMB genes localized on chromosome 6

**Fig. 1:**
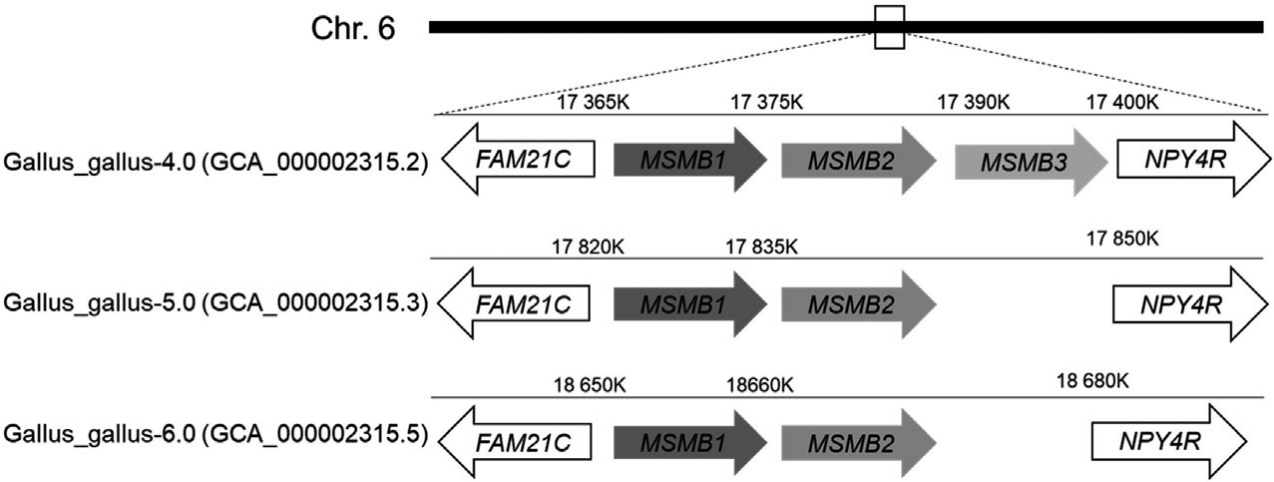
The annotation of MSMB3 gene on chicken chromosome 6 remains controversial. Information related to the genomic localization of chicken MSMB paralogs was extracted from Ensembl (www.ensembl.org). Chicken MSMB3 gene (LOC101750704) was withdrawn from the two last chicken genome assemblies (Gallus_Gallus‐5, GCA_000002315.3 and Gallus_Gallus‐6, GCA_000002315.5) but was reported to co‐localize with MSMB1 (LOC101750594) and MSMB2 (LOC100858647) paralogs in the older Gallus_Gallus‐4 assembly (GCA_000002315.2).

The exact localization of MSMB1, MSMB2, and MSMB3 on chicken chromosome 6 remains controversial. MSMB1 (LOC101750594) and MSMB2 (LOC100858647) are co‐localized within a 30 to 35 kb locus on chromosome 6 (6:18655953–18660462 and 6:18666862–8670817), regardless of the genome assembly version. However, MSMB3 was identified in *Gallus_gallus* 4.0 assembly but was withdrawn in the latest genome assemblies (5.0 and 6.0; Fig. 1), due to lack of supporting evidence in the current genome build. But using Batch Coordinate Conversion/liftOver [36], the genomic region corresponding to MSMB3 gene in *Gallus_gallus* 4.0 assembly (chr6:17390530–17392831) could still be identified in the genomic region corresponding to chr6:18677529–18679830 in *Gallus_gallus* 6.0 assembly/GCA_000002315.5, but lacks annotation in the assembly currently available.

### The protein sequence of chicken MSMB3 is highly conserved in avian species

**Fig. 2:**
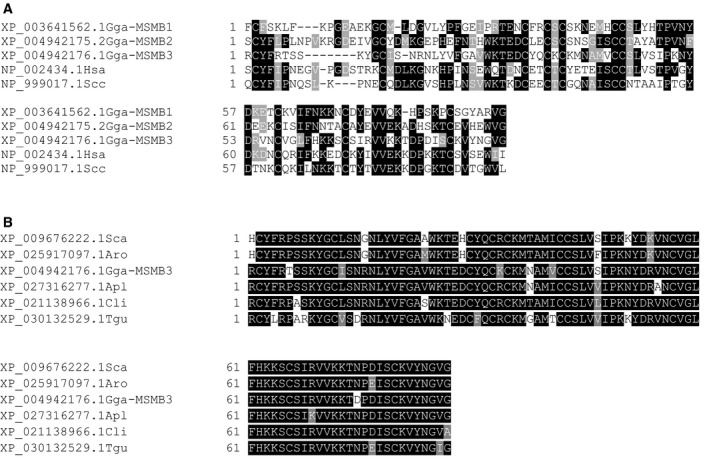
Multiple sequence alignment of chicken MSMB protein. (A) Protein sequence alignment of chicken MSMB3 with chicken MSMB1 and MSMB2 paralogs, and human and porcine MSMBs. (B) Sequence alignment of mature MSMB3 from different bird species. Palaeognathae subclass (flighless lineages ratites) is represented by the Southern African ostrich (*Struthio camelus australis*, Sca, Struthioniformes) and Okarito kiwi (*Apteryx rowi*, Apr, Apterygiformes). Neognathae subclass is illustrated by Galloanserae (clade of fowls, including red junglefowl chicken, *Gallus gallus*, Gga, Galliformes, and mallard duck, *Anas platyrhynchos*, Apl, Anseriformes), and Neoaves (clade of modern birds, with rock dove, *Columba livia*, Cli, Columbiformes, and zebra finch, *Taeniopygia guttata*, Tgu, Passeriformes). Alignments were performed using T‐Coffee (http://www.ebi.ac.uk/Tools/msa/tcoffee/) with BLOSUM62 matrix and formatted with BOXSHADE (http://www.ch.embnet.org/software/BOX_form.html). Identical and similar residues are indicated by black and gray boxes, respectively.

The comparison of protein sequences of the three chicken paralogs indicates a moderate sequence identity ranging from 34% (MSMB2 and MSMB3) to 42% (MSMB1 and MSMB2; Fig. 2A). Among chicken paralogs, chicken MSMB2 possesses the highest percentage of sequence identity with human and porcine MSMBs (46.8% and 46.1%, respectively; Fig. 2A) as well as conserved motifs such as DXKG, HXXN, ISCC, VVEKXD, KTC, CYFXP, and the last W residue that are not recovered in chicken MSMB1 and MSMB3. These observations suggest that chicken MSMB2, porcine, and human MSMBs may be orthologous genes. As expected, using BlastP program [37], the highest sequence similarities with chicken MSMB3 were found with proteins from bird species. Figure 2B illustrates the alignment of six sequences of avian MSMB3 encompassing Neognathae (two Galloanserae and two Neoaves) and Palaeognathae (including two flightless lineage ratites) subclasses. The percent identity matrix comparing these avian MSMB3 sequences reveals that they share at least 80% sequence identity, which indicates that this protein is highly conserved among bird species. To our knowledge, there are no MSMB3 orthologs in nonavian species.

### Chicken MSMB3 is essentially expressed in the magnum tissue that is responsible for egg white formation

**Fig. 3:**
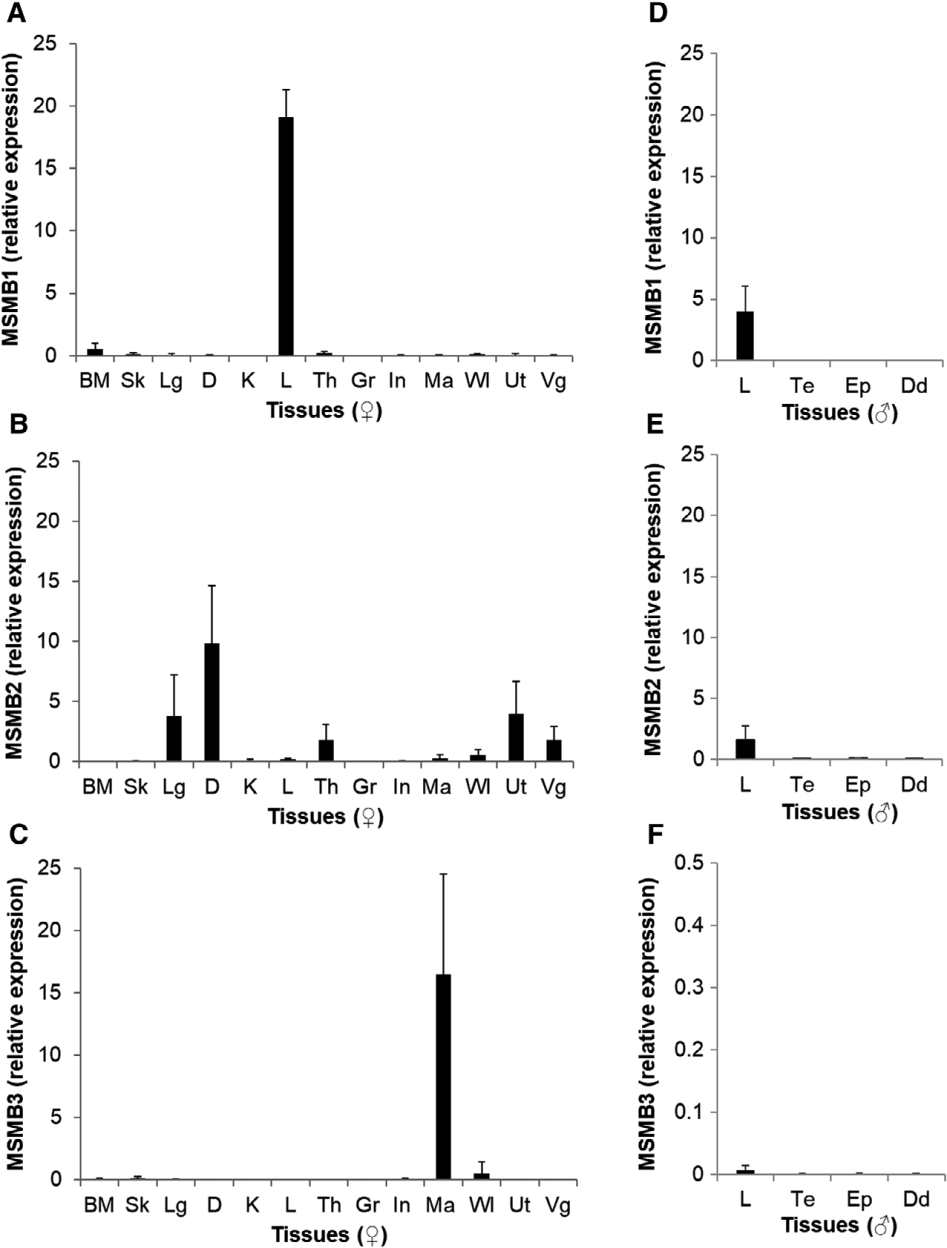
Expression of chicken MSMB proteins in chicken tissues. The expression of chicken MSMB1, MSMB2, and MSMB3 was investigated in a broad range of chicken female tissues (panels A, B, and C, respectively) and in the liver and reproductive tissues of chicken males (panels D, E, and F, respectively). Data are presented as mean ± standard deviation (*n* = 4–8, depending on the number of biological replicates available). Note that *y*‐axis scale for panel F is highly reduced compared with the other panels (0–0.5 relative expression versus 0–25). BM, bone marrow; D, duodenum; Dd, ductus deferens; Ep, epididymis; Gr, granulosa cells; In, infundibulum; K, kidney; L, liver; Lg, lung; Ma, magnum; Sk, skin; Th, theca; Ut, uterus; Vg; vagina; WI, white isthmus.

The expression of chicken MSMB genes was analyzed in various reproductive and nonreproductive tissues of hens (adult female chickens), in the liver, and in reproductive tissues of roosters (adult male chicken), to appreciate their tissue specificity. MSMB1 gene is almost exclusively expressed in the liver of both male and female chickens (Fig. 3A,D), while it is barely detectable in reproductive tissues. Among the three chicken MSMB genes, MSMB2 seems to have the broader tissue expression pattern, as its expression is detected in several tissues [duodenum, lung, female reproductive organs, including theca, magnum, white isthmus, uterus, and vagina (Fig. 3B)]; however, a very weak expression of MSMB2 is observed in the male reproductive tract (Fig. 3E). In contrast to the two other MSMB paralogs, MSMB3 is expressed in the magnum and to a lesser extent in the white isthmus, which are involved in the secretion of proteins composing the egg white and the eggshell membranes, respectively (Fig. 3C). It is noteworthy that the expression of MSMB3 is barely detectable in male tissues (Fig. 3F).

### Mass spectrometry analysis unveiled the presence of MSMB3 homodimer

Chicken egg MSMB3 purified from egg white was analyzed by top‐down proteomics using matrix‐assisted laser desorption ionization–time of flight (MALDI‐TOF) mass spectrometry and micro‐liquid chromatography coupled to high‐resolution mass spectrometry (µLC‐MS).

**Fig. 4:**
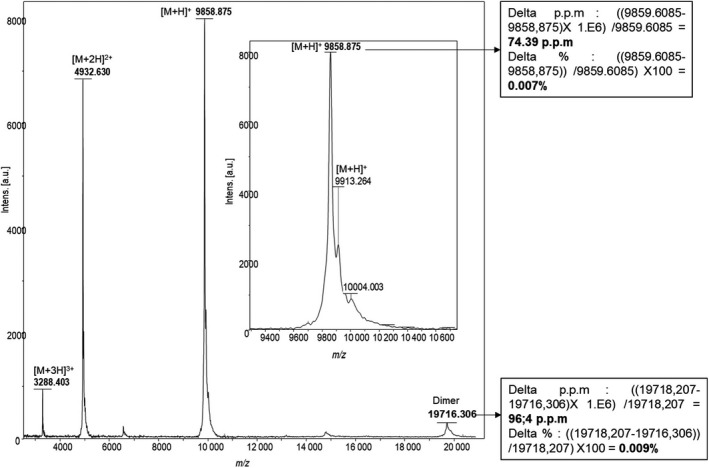
MALDI‐TOF spectra of chicken MSMB3. MALDI‐TOF spectrum of the chicken MSMB3 showing 4 *m*/*z* major peaks. The major *m*/*z* peak at 9858.875 corresponds to the monocharged form [M + H]^+^ of chicken MSMB3 sequence without G residue and, including five disulfide bridges (theoretical average mass at 9859.60 Da). The other peaks at *m*/*z* 4932.630, 3288.403, and 19716.306 correspond to multicharged forms as [M + 2H]^2+^, [M + 3H]^3+^, and dimer, respectively. Insert: focus on MSMB3 monocharged proteoform [M + H]^+^, including a minor peak related to the intact/native chicken MSMB3 proteoform (*m*/*z* = 9913.263).

Firstly, top‐down analysis was performed without any reduction or alkylation in order to characterize whole and intact proteoforms. The MALDI‐TOF spectrum of the MSMB3 fraction showed several *m*/*z* peaks related to MSMB3 protein (Fig. 4). Interestingly, the major *m*/*z* peak at 9858.88 is not consistent with the theoretical [M + H]^+^ average mass of MSMB3, considering the five disulfide bridges (9916.66, Table 1). This value would rather coincide with the monocharged average mass of MSMB3 sequence with five disulfide bridges, lacking a G residue at its carboxy‐terminal extremity (MSMB3(‐G)) that displays a theoretical average mass at 9859.61 Da (Table 1). The other peaks at *m*/*z* 4932 and 3288 correspond to multicharged forms as [M + 2H]^2+^ and [M + 3H]^3+^, respectively. However, a peak of minor abundance at 9913.26 *m*/*z* corresponding to the whole chicken MSMB3 sequence (with the G terminal and five cysteines) remains detectable (theoretical average mass at 9916.66 Da; Fig. 4, insert). In addition, it is noticeable that the peak at 19716.31 *m*/*z* is consistent with a dimer of MSMB3(‐G).

**Table 1:** Theoretical monoisotopic and average masses for MSMB3 proteoforms and corresponding MSMB3 dimers

Accession number	Protein sequence	[M + H]^+^ average	[M + H]^+^ monoisotopic	[M + H]^+^ average with 5 disulfide bridges	[M + H]^+^ monoisotopic with 5 disulfide bridges	Dimer [2M + H]+	Dimer [2M + H]^+^ with 10 disulfide bridges
>XP_004942176.1 (*Gga*‐MSMB3)	RCYFRTSSKYGCISNRNLYVFGAVWKTEDCYQCKCKMNAMVCCSLVSIPKNYDRVNCVGLFHKKSCSIRVVKKTDPDISCKVYNGVG	9926.7398	9919.7874	9916.6604	9909.7091	19852.4796	19832.3208
>XP_004942176.1 (*Gga*‐MSMB3 without G)	RCYFRTSSKYGCISNRNLYVFGAVWKTEDCYQCKCKMNAMVCCSLVSIPKNYDRVNCVGLFHKKSCSIRVVKKTDPDISCKVYNGV	9869.6879	9862.7659	9859.6085	9852.6877	19738.3758	19718.207

**Fig. 5:**
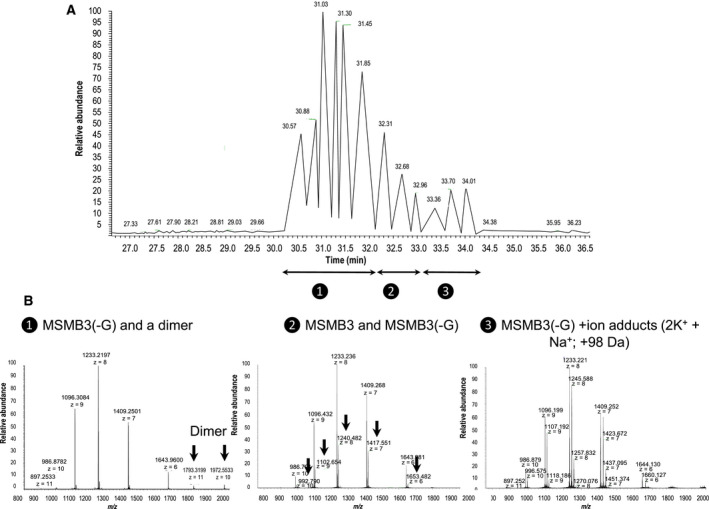
µLC‐MS of chicken MSMB3. (A) Representative chromatogram obtained by µLC‐MS. B. Combined spectra from chromatographic peaks revealing the presence of MSMB3(‐G) and a dimer (❶), intact MSMB3 and MSMB3(‐G) proteoforms (❷), and all proteoforms with sodium and potassium ions adducts (❸).

The chicken egg MSMB3 fraction was further analyzed by µLC‐MS to characterize intact MSMB3 proteoforms with a better mass accuracy (< 100 p.p.m. as observed by MALDI‐TOF). From the chromatogram (Fig. 5A), combined spectra obtained between 30 and 32, 32 and 33, and 33 and 34 min of retention time ranges were allowed to characterize the MSMB3(‐G) and the dimer, the MSMB3(‐G) and MSMB3, all proteoforms with salt adducts that include one sodium and two potassium ions, respectively (Fig. 5B1–B3). On the basis of the high‐resolution mass spectra, the calculated mean monoisotopic mass [M + H]^+^ is 9852.70 (± 1.50 p.p.m.) for chicken MSMB3(‐G) and 9910.71 (± 0.29 p.p.m.) for intact chicken MSMB3 (Figs S1 and S2, respectively). The average mass of the chicken MSMB3 dimer was observed at *m*/*z* 1793.3199 (z = 11) and *m*/*z* 1972.5533 (*z* = 10; Fig. 5B1) corresponding to [M + H]^+^ at 19716.51 and 19716.53 Da. These data corroborate MALDI‐TOF observed results but are not in agreement with the theoretical mass of 19718.20 (delta mass of about 2 Da). The presence of the two proteoforms was also evidenced by a bottom‐up proteomic approach. MSMB3(‐G) and MSMB3 were identified with 94% and 87% of sequence coverage, respectively, each with about 20 peptides. Expected modifications as carbamidomethylation on cysteines or oxidation of methionines were detected. However, modification with a delta mass of −1 Da on two lysines and especially on K63 corresponding to a potential oxidation of the lysine residue was identified (Fig. S3). These mass deviations may explain the delta mass of 2 Da that was observed for the homodimer by MALDI‐TOF and µLC‐MS (see previous paragraph).

### MSMB3 homodimer is confirmed in solution by NMR experiments

The homodimeric state of MSMB3 in solution was investigated by NMR spectroscopy. First, we checked the sample quality by one‐dimensional ^1^H NMR and 2D ^1^H total correlation spectroscopy (TOCSY; Fig. S4A,B, respectively). The 2D ^1^H TOCSY spectrum showed good dispersion in the amide region, which indicates a highly structured protein. ^1^H^α^ chemical shifts observed in the 4.5–5.7 p.p.m. region support that the protein is mainly in a β‐sheet conformation. However, two types of correlation peaks coexisted in the amide region: Some are very thin and intense, and some are very broad. This feature can be related to an exchange process at the NMR intermediate timescale, which can be due to conformational changes within the protein, or to a monomer–dimer exchange that can only be detected on the residues involved at the dimer interface. Determination of the translational diffusion coefficient from pulsed‐field gradient NMR experiments (DOSY) enabled us to determine that the protein exhibited a diffusion coefficient of 1.29 10^−10^·m^2^·s^−1^. As compared with the diffusion coefficient values found in the literature (Table S1), this value is compatible with a dimeric form of the protein. Since high concentration could stabilize a dimeric conformation, the sample was then diluted to a 50 μm concentration. No quantitative impact on the relative intensities of the peaks was observed on the 2D TOCSY experiment and on the measured diffusion coefficient. To ensure that the dimeric conformation is present in solution, we built a calibration curve (Fig. S4C) and compared it to the same curve obtained with the values from the literature (Fig. S4D). Both value sets can be represented by the power law 3 10^−9^Mw^−0.344^ and 3 10^−9^Mw^−0.347^ with a R^2^ of 0.997 and 0.976, respectively. As shown in Fig. S4C, the measured diffusion coefficient for the MSMB3 is on the calibration curve for a molecular mass corresponding to a dimeric state of the protein. It clearly supports that MSMB3 protein adopts a dimeric conformation in solution.

### The X‐ray structure of chicken MSMB3 shows the homodimer structure and reveals subtle structural differences compared with MSMBs from nonavian species

**Fig. 6:**
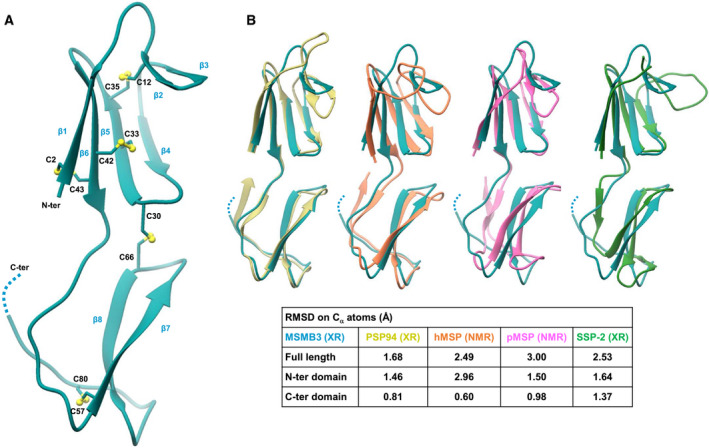
X‐ray 3D structure of chicken MSMB3. (A) Overview of MSMB3 X‐ray structure. (B) Superimposition of chicken MSMB3 (blue) and MSMB homologs in human by X‐ray and NMR (PSP94, PDB:3ix0 [12], and hMSP, PBD:2iz3 [9], in yellow and orange, respectively), in pig by NMR (pMSP, PDB: 2iz4 [9], pink), and in snake by X‐ray (SSP‐2, PDB:6imf [10], green) with table summarizing RMSDs of chicken MSMB3 structure and published MSMB structures.

The X‐ray 3D structure of MSMB3 purified from chicken egg white was solved at 2.14 Å resolution (Table 2). We observe the presence of two molecules in the asymmetric unit (named monomer A and monomer B) sharing a similar fold (RMSD = 1.4 Å on all Cα atoms). MSMB3 consists of two domains (amino‐ and carboxy‐terminal) linked together by a linker peptide. The spatial arrangement of the two domains is likely to be stabilized by the disulfide bridge (C30–C66; Fig. 6A). The amino‐terminal domain (residues 1–45) possesses a four‐stranded antiparallel β‐sheet (β1, β4, β5, and β6), which topological structure corresponds to a Greek key motif, and a small two‐stranded β‐sheet (β2 and β3) inserted between β1 and β4 strands. The four‐stranded β‐sheet is stabilized by two disulfide bonds that connect the β6 strand to β1 (C2–C43) and β5 (C33–C42). A third disulfide bond (C12–C35) contributes to constrain the small two‐stranded β‐sheet (β2 and β3). The carboxy‐terminal domain (residues 46–86) has one antiparallel β‐sheet composed of two strands (β7 and β8) and one disulfide bond (C57–C80) linking the carboxy‐terminal residues to the β‐sheet. Electron density was missing for the last residues G85 and V86 of MSMB3(‐G) likely due to their high mobility.

**Table 2:** X‐ray data collection and refinement statistics.

Data collection statistics	
Radiation source	ESRF ID29
Wavelength (Å)	0.97903
Space group	P2_1_2_1_2
Cell dimensions: *a*, *b*, *c* (Å)	47.18, 101.05, 36.91
Resolution range (Å)	42.75–2.14 (2.18–2.14)
Total observations	56 667 (1227)
Unique reflections	10 095 (446)
Completeness (%)	98.8 (91.6)
Multiplicity	5.6 (2.8)
*R*_merge_^a^ (%)	8.2 (53.6)
Average *I*/*s*(*I*)	12.0 (1.6)
CC_1/2_ (%)	99.8 (72.4)
Refinement and model statistics	
Resolution range (Å)	42.75–2.14
Number of reflections used	10 093
*R*_work_^b^/*R*_free_^c^ (%)	22.1/24.3
Average *B* values (Å^2^)	
All atoms	47.42
Protein chain A atoms	43.77
Protein chain B atoms	49.44
${\text{SO}}_{4}^{2‐}$ atoms	63.08
Citrate atoms	84.50
Ethane‐1,2‐diol atoms	65.91
Pentaethylene glycol	75.95
Water atoms	44.45
Root mean square deviation from ideality	
Bond lengths (Å)	0.002
Bond angles (°)	0.420
Ramachandran analysis (% of residues)	
Favored regions/allowed regions/outliers	96.3/3.7/0.0
Number of atoms	
Protein chain A	652
Protein chain B	631
${\text{SO}}_{4}^{2‐}$	15
Citrate	13
Ethane‐1,2‐diol	4
Pentaethylene glycol	16
Water	46

a, b, c are the lengths of basic vectors, lengths of cell edges in angstroms. As P21212 belongs to orthorhombic space group, angles of the cell are 90° and are not mentioned. Unit cell parameters are usually mandatory in publications.

**Fig. 7:**
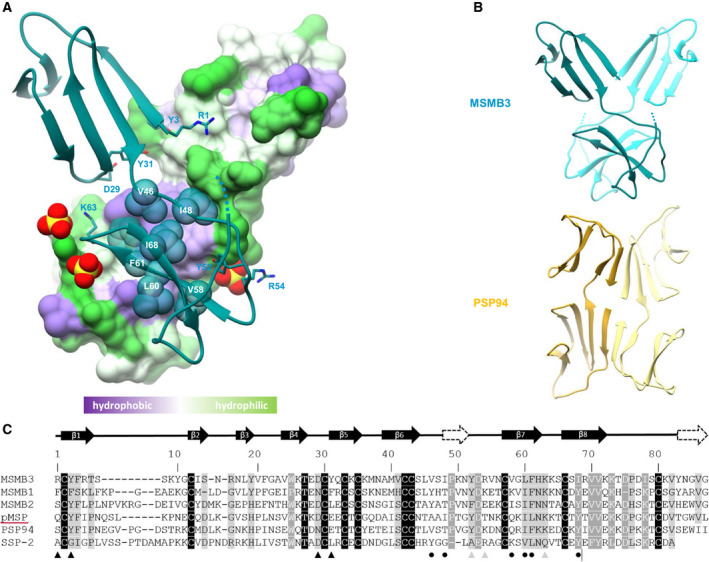
Binding interface of a predicted MSMB3 dimer. (A) 3D structure of the MSMB3 homodimer. Monomer A is shown as a blue ribbon. Monomer B is shown as a molecular surface colored according to the values of hydrophobic potentials. Sulfate ions are indicated using a Corey–Pauling–Koltun (CPK) representation in red (oxygen atom) and yellow (sulfur atom). (B) Overview of chicken MSMB3 (top) and human PSP94 (bottom) dimers. Monomer A and monomer B of MSMB3 are shown in dark blue and light blue, respectively. Monomers A and monomer B of PSP94 are shown in dark yellow and light yellow, respectively. (C) Sequence alignment of chicken MSMB1, MSMB2, MSMB3, porcine pMSP, human PSP94, and Viperidae snake SSP‐2 with assigned secondary structures. The two β‐strands present in PSP94 and not in MSMB3 are shown as dashed arrows. In chicken MSMB3 structure, residues connecting sulfate ions are marked with a gray triangle, residues making interchain H‐bonds are marked with a black triangle, and residues of the hydrophobic core are illustrated by a black dot.

The 3D structure of chicken MSMB3 is globally similar to human (PSP94, hMSP) and porcine (pMSP) MSMBs (RMSD = 1.68, 2.49, and 3.00 Angströms, respectively; Fig. 6B). The main difference resides in the carboxy‐terminal domain in which only one double‐stranded β‐sheet is present in MSMB3, whereas the human and the porcine proteins have two double‐stranded antiparallel β‐sheets. This missing β‐strand is due to the predicted mobility of the last two MSMB3 residues G85 and V86 (G87 is missing in MSMB(‐G)). However, the last three residues of PSP94 carboxy‐terminal extremity are W92, I93, and I94 with W92 (W85, I86, and I87 in Fig. 7C) lying over the top of two hydrophobic residues (P56 and V89). This particularity is assumed to stabilize the carboxy‐terminal residues of PSP94. Despite the difference in the number of secondary structures, the fold of the carboxy‐terminal domain of MSMB3 is conserved between species (Fig. 6B). The absence of this β‐sheet in MSMB3 structure is also seen for Viperidae snake small serum protein 2 (SSP‐2), which has a shorter carboxy‐terminal domain than the other members of the MSMB family.

As two MSMB3 molecules were present in the asymmetric unit of the MSMB3 crystals, we investigated the protein quaternary assembly using the PISA server (https://www.ebi.ac.uk/pdbe/pisa/). Results show that monomer A and monomer B may form a stable homodimer in solution with a buried area of 867 Å^2^ that is stabilized by a total of eight H‐bonds, including one salt bridge. The two monomers are held together mainly via their respective carboxy‐terminal domains, primarily through a cluster of hydrophobic residues (V46, I48, V58, L60, F61, and I68; Fig. 7A). Four H‐bonds are detected between the main chain atoms of residues G59 and F61. Another H‐bond between atoms P49(A) O and K63(B) Nζ reinforces the homodimer stability. Concerning the amino‐terminal part of the dimer, fewer contacts are found between these two domains. Two H‐bonds involve the residues Y31 and Y3, and one salt bridge is found between residues R1(A) and D29(B) (Fig. S5). Most of those residues are well conserved within the MSMB3 family but not in other MSMB proteins (Figs 2 and 7C).

The MSMB3 dimer observed here is different from the one seen in PSP94 crystals (Fig. 7B,C). In PSP94, the dimer buried area is about 930 Å^2^ and the dimeric association requires the β10 strand, which is absent in MSMB3. In addition, three sulfate ions arising from the crystallization conditions and bridging the carboxy‐terminal domains of the two monomers were observed in the electron density maps (Fig. S6). Sulfate ions 101(A) and 101(B) are linked to residues K63 and K64 of one chain and to R54 of the other chain in a symmetric fashion. Furthermore, the third sulfate ion 102(A) is located close to the 101(A) sulfate ion and makes contact with the side chain of R54(B) residue.

### The chicken MSMB1 and MSMB2 models (built by homology modeling) display distinct electrostatic potentials compared with chicken MSMB3

**Fig. 8:**
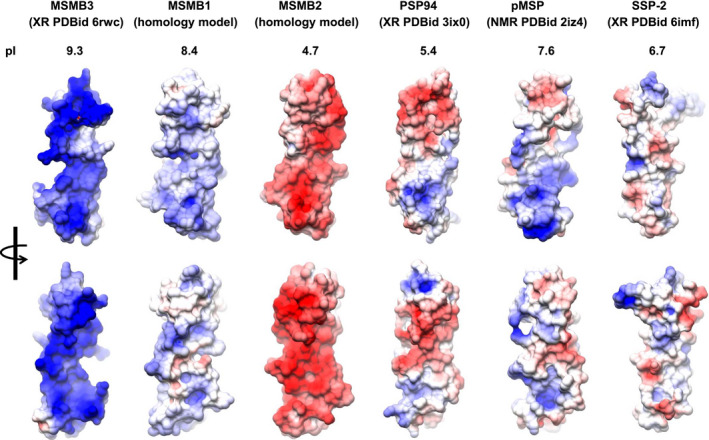
Molecular surface of various MSMB structures (monomers) colored according to electrostatic potential values. Electrostatic potentials were calculated using the APBS server (http://www.poissonboltzmann.org/) [59, 60] using default parameters. The same electrostatic potential energy scale was used for all representations in Chimera. Upper orientations are the same as in Fig. 6. Red, negative potential; white, neutral potential; blue, positive potential. Theoretical pI values were calculated using ProtParam tool of the expasy server (https://web.expasy.org/protparam/) using sequences extracted from PDB files.

Because the calculated isoelectric point (pI) values of the three chicken paralogs are very different (pI = 4.7 for MSMB2, pI = 8.4 for MSMB1, and pI = 9.3 for MSMB3), we expect differences in the electrostatic distribution at the molecular surface of these proteins. To achieve this comparison and because the 3D structures of MSMB1 and MSMB2 were not available, we built the 3D models of both proteins by homology modeling using X‐ray MSMB3 as the template. Figure 8 illustrates the solvent‐accessible surface of MSMB proteins colored according to electrostatic potential values (blue: positive charges; red: negative charges). Unlike PSP94, pMSP, and SSP‐2 where the positive and negative charges are clustered, the positive charges of MSMB3 and the negative charges of MSMB2 are evenly distributed on the protein surfaces. To a lesser extent as compared with MSMB3, MSMB1 also exhibits an even distribution of positive charges at its molecular surface.

## Discussion

Microseminoprotein proteins are widely distributed in the animal kingdom and display a broad range of biological activities. MSMB genes are present in one single copy in most mammals, while multiple copies have been identified in marsupials (14 paralogs), zebrafish (three paralogs), and chicken (three paralogs) [1, 4]. The biological significance of these numerous MSMB proteins in some species is still obscure.

Chicken MSMB3 paralog has been recently purified, and its protein sequence has been corroborated by mass spectrometry [26]. However, the genomic localization of the related gene remains controversial because of the withdrawal of MSMB3 gene in the two last chicken genome assemblies (Fig. 1). This inconsistency will need to be further corrected as the absence of this chicken MSMB3 in protein databases, including the National Center for Biotechnology Information databank, may introduce some bias when performing proteomics on egg‐derived samples. Therefore, the presence and relative abundance of chicken MSMB3 in chicken egg may have been underestimated in the last decade (corresponding to the release of chicken genome assemblies 5.0 and 6.0). The present article provides further compelling evidence that MSMB3 gene and protein product both exist in the chicken species and, likely, in many other avian species. Indeed, this work reveals not only that MSMB3 orthologs are present in other bird species, including Palaeognathae and Neognathae with a high percentage of protein sequence identity (at least 80%, Fig. 2B), but also that MSMB3 gene is highly expressed in the chicken oviduct (Fig. 3) related to egg formation and avian reproduction. More importantly, MSMB3 protein has been purified from chicken egg white (this report and [26]), its sequence with several modifications has been characterized by proteomics, and its X‐ray 3D structure has been solved (Figs 6 and 7).

The three chicken paralogs MSMB1, MSMB2, and MSMB3 share relatively low percentage of sequence identity (34.5% between MSMB2 and MSMB3, 40.7% between MSMB1 and MSMB3, and 42% between MSMB1 and MSMB2), which is supposed to affect their respective biological functions. To better appreciate the specificities of each chicken MSMB genes, we first investigated their relative expression in various chicken female and male tissues. We show that all three chicken paralogs have a distinct pattern of expression with MSMB1 gene being essentially expressed in the liver, MSMB2 displaying a more ubiquitous expression, while MSMB3 being almost exclusively expressed in the female reproductive tissue (magnum) that secretes egg white proteins. The high expression of MSMB1 in the liver of both male and female chickens suggests a crucial physiological role in the liver metabolism that, however, remains to be defined. Concerning MSMB2, the detectable (although low) expression of this paralog in the testis, ductus deferens, and epididymis (Fig. 3E) of chickens is in accordance with the identification of MSMB2 protein in male reproductive secretions [31]. Thus, chicken MSMB2 may have a role in chicken male reproduction, similarly to the human homolog PSP94. However, it is noteworthy that MSMB2 is more widely expressed than human PSP94 whose expression is specific to the prostate tissue [38]. The expression of chicken MSMB2 in the white isthmus, the uterus, and vagina, which is corroborated by the identification of this protein in the eggshell and eggshell membranes [39, 40], also suggests a function in eggshell formation and structure. Finally, MSMB2 was shown to be highly expressed in the duodenum, lung (Fig. 3B), and to a lesser extent in the liver (Fig. 3B,E), and consequently, it may have other physiological functions, besides reproduction. Compared to MSMB1 and MSMB2, MSMB3 is merely expressed in the reproductive tissues of female chickens, especially in the magnum (strong expression) and in the white isthmus (low expression), which is in accordance with the identification of this MSMB3 protein specifically in egg white and eggshell membranes, respectively [26, 41]. The tissue specificity of MSMB3 strongly supports a role in chicken embryonic development. Recently, a *msmb3* gene from Xenopus was described as a potent regulator of neural crest migration [35]. However, the mature form protein corresponding to Xenopus *msmb3* gene as identified in databank only shares 38.37% sequence identity with mature chicken MSMB3, while it shows 47.67% and 46.67% sequence identity with chicken MSMB1 and MSMB2, respectively. Such an observation suggests that Xenopus *msmb3* gene is rather orthologous to chicken MSMB1 or MSMB2 genes. Because of its antibacterial activity, we have previously suggested that chicken MSMB3 may participate in the protection of the embryo [26, 32], together with the numerous antibacterial proteins and peptides that have been identified in the egg [42]. Interestingly enough, this protein persists in the egg even in the late stages of embryonic development [32], when the egg white is transferred into the amniotic fluid to be subsequently swallowed by the embryo. This intriguing MSMB3 stability throughout embryonic development is likely due to the presence of numerous protease inhibitors in the egg white [43] that prevent proteolysis, but also to the predicted resistance of disulfide‐rich proteins to proteolysis [44]. The fate of MSMB3 protein, once orally absorbed by the embryo, remains unknown, but it may participate in the gut immunity of chicks as long as it resists degradation by digestive enzymes [32]. To conclude, the very diverse profile of expression of all three paralogs is assumed to reflect diverging functions, which is further supported by the moderate percentage of sequence identity between all three paralogs (Fig. 2A).

Chicken MSMB3 was purified from egg white using heparin affinity chromatography and exclusion chromatography [26] prior to mass spectrometry, in‐solution NMR spectroscopy, and crystallization for X‐ray diffraction analysis. Mass spectrometry analyses reveal that the major form of purified MSMB3 lacks the C‐terminal Gly residue at the carboxy‐terminal extremity, although the whole form remains detectable at a much lower abundance (Figs 4 and 5). The hydrolytic mechanism underlying this glycine removal is not known yet. Indeed, only few proteases and peptidases at a very low abundance were identified in the egg white, and none of them possess the substrate specificity required to cleave this carboxy‐terminal residue (aminopeptidase Ey, renin, similar to transmembrane protease, serine 9, similar to carboxypeptidase D, similar to aminopeptidase A, aminopeptidase) [45]. In addition, the presence of very abundant protease inhibitors restricts such proteolytic events in egg white [43]. In the future, it might be interesting to analyze whether secreted chicken MSMB1 and MSMB2 also lack the carboxy‐terminal glycine.

In addition to the post‐translational modification of MSMB3 sequence, a *m*/*z* peak of low amplitude corresponding to a MSMB3 potential homodimer could be detected on mass spectra (Figs 4 and 5). It is noteworthy that such a technique usually triggers dissociation of noncovalent protein complexes. Thus, this molecular species is likely underestimated. The presence of a dimer was further confirmed by analyzing NMR spectroscopy data and by X‐ray diffraction. Both approaches concurred to conclude that the major form of MSMB3 is a homodimer. It is not known whether chicken MSMB1 and chicken MSMB2 are also prone to similar homodimerization arrangement, considering their relatively low sequence identity with chicken MSMB3 (Fig. 2A).

The 2.14 Å crystal structure of chicken MSMB3 reveals that the monomer adopts a fold that resembles those determined for human, porcine, and snake MSMBs. Chicken MSMB3 consists of an amino‐terminal domain having a Greek key fold and a double‐stranded beta‐sheet carboxy‐terminal domain held together by a disulfide bond. Compared with human and porcine MSMBs, chicken MSMB3 lacks the last β‐sheet within the C‐terminal domain, likely due to the high mobility of its carboxy‐terminal residues (G85, V86). This absence of the two beta‐strands in MSMB3 results in a different pattern of dimerization compared with human MSMB (PSP94) where the two monomers endorse an edge‐to‐edge association ([12], Fig. 7B). Although MSMBs can be found as homodimers in solution, it is commonly assumed that the monomeric proteins are believed to be the relevant biologically active species [46]. As an example, the monomeric PSP94 protein was found to interact with monoclonal natural killer‐associated anti‐Leu‐111b antibody, whereas the dimeric form was inactive [47, 48]. Moreover, the 3D structure of the SSP‐2‐triflin complex revealed that the venom inhibitor SSP‐2 interacts as a monomer with the cysteine‐rich secretory protein [10].

When comparing the distribution of the negative and positive charges at the surface of chicken MSMB3 protein with the other available MSMB proteins, we observed a very different pattern of charge distribution on chicken MSMB3. Furthermore, chicken MSMB3 protein is characterized by a high cationicity (pI = 9.3), as opposed to human, porcine, and snake MSMBs (acidic or neutral pI). Using chicken MSMB1 and MSMB2 structural models, we also showed that this feature is specific to the MSMB3 paralog: The distribution of positive charges onto MSMB1 model is more diffused despite a cationic pI (pI = 8.4), while the surface of MSMB2 model (pI = 4.7) is essentially anionic. Such a high cationicity of the chicken MSMB3 protein together with the presence of numerous clusters of positive charges may explain its affinity to heparin, a negatively charged glycosaminoglycan [26]. In addition, the peculiar conformation of MSMB3 homodimer may also contribute to form additional conformational positive clusters to enhance heparin binding. Fortunately, MSMB3 was crystallized using buffer containing sulfate ions, which could be easily assigned into electron density maps. The X‐ray 3D structure revealed that residues R54, K63, and K64 are involved in H‐bonds with the sulfate ions, which suggests that these residues might be important for heparin binding and activity. Knowing that glycosaminoglycans are tightly associated with extracellular matrix proteins and cells, the affinity of MSMB3 for glycosaminoglycans may reinforce the hypothesis of a role during embryonic development [33]. It might also play a role in the progression of extra‐embryonic structures (such as cell migration), namely the yolk sac, onto the inner surface of the perivitelline layer that encloses the yolk [49].

## Concluding remarks

To conclude, given its specific expression in the female reproductive oviduct, its physiochemical and structural specificities, and its high sequence conservation among birds, MSMB3 protein is believed to play a crucial role in the reproduction of avian species. The next challenges will be to decipher its exact underlying physiological functions and regulation during incubation of fertilized eggs and to investigate whether all three paralogs are expressed during embryonic development, by the embryo itself and/or by the extra‐embryonic annexes. The divergent features of chicken MSMB paralogs and the selectivity of their tissue expression that are likely associated with a specific function might motivate further studies on the relationships between structure of MSMB proteins and their respective activity in vertebrates.

## Methods

### Purification of MSMB3 from egg white

Briefly, egg whites were collected from freshly laid eggs (Isa‐Hendrix, St Brieuc, France). Egg whites were homogenized, sampled, and kept frozen until further use. Chicken egg MSMB3 was further purified by heparin affinity chromatography followed by gel filtration, as described previously [26]. The protein concentration of MSMB3 was measured using absorbance at 280 nm, considering its specific extinction coefficient (E1% = 15.18) [26]. The purity of purified MSMB3 was assessed by SDS/PAGE and mass spectrometry analysis.

### Sequence analyses

Alignments were performed using T‐Coffee (http://www.ebi.ac.uk/Tools/msa/tcoffee/) with BLOSUM matrix and formatted with BOXSHADE (https://embnet.vital‐it.ch/software/BOX_form.html). Theoretical pI values were extracted from ProtParam tool (https://web.expasy.org/protparam/).

### Chicken handling and housing

Eight hens (Isabrown; Isa‐Hendrix) were bred at PEAT INRAE Poultry Experimental Facility (2018, https://doi.org/10.15454/1.5572326250887292E12). Animals were housed and fed according to recommendations defined by the ‘Institut national de recherche pour l’agriculture, l’alimentation et l’environnement, (INRAE)’, France.

### mRNA extraction and real‐time quantitative PCR

Tissues were collected from eight 60‐week‐old laying hens. Animals were euthanized with Dolethal® (Vetoquinol, Magny‐Vernois, France). Tissues were harvested from the oviduct (infundibulum, magnum, white isthmus, uterus, vagina), ovary (granulosa, theca), and other organs (lung, liver, kidney, bone marrow, skin, and duodenum). The tissues were snap‐frozen in liquid nitrogen and stored at −80 °C. Male tissues (liver, testis, ductus deferens, and epididymis), collected from 40‐week‐old roosters (Hubbard, Quintin, France; Novogen, Loue, France), were a kind gift from A. Thelie (PRC, INRAE, CNRS, IFCE, Université de Tours, Nouzilly, France).

For female tissues, total RNA was extracted from frozen tissues using nucleospin RNA II (Macherey‐Nagel, Düren, Germany) for infundibulum, magnum, white isthmus, and kidney, RNAL (Macherey‐Nagel) for uterus and liver, and the RNA NOW method (Biogentex, Ozyme, Saint Quentin en Yvelines, France) for lung, duodenum, vagina, granulosa, theca, and skin. Bone marrow RNA was extracted by the double extraction method TRI Reagent and TRI Reagent LS (Sigma‐Aldrich, St Louis, MO, USA). Samples were treated with DNase I (Applied Biosystems, Courtaboeuf, France). RNA concentrations were determined by measuring the absorbance at 260 nm, and the quality of RNA was assessed using the Agilent 2100 Bioanalyzer System (Agilent Technologies, Massy, France). Total RNA (5 µg) was reverse‐transcribed using the Superscript II Kit (Invitrogen, Cergy Pontoise, France) and oligo(dT) (Promega, Madison, WI, USA), and stored at −80 °C until further use. Total RNA and cDNA from liver, ductus deferens, epididymis, and testis from breeder males were prepared as previously described [50].

Primers for ‘beta‐MSMB‐like’ (MSMB1, LOC100858647, XM_003641514.3, Fw‐ATTGTTCAAGCCAGGAGAGGC; Rev‐TTCACAGGAGTGTGATAGAGGG), for ‘beta‐MSMB A1‐like’ (MSMB2, LOC101750594; XM_004942118.2; Fw‐TGGTGATGAGATTGTAGGCTGC; Rev‐TGCCACTCCTTTGTTAGCCC), and for ‘beta‐MSMB‐like’ (MSMB3, LOC101750704; XM_004942119.1; Fw‐TTCTCTAGGACCCTGTGCGAT; Rev‐GTCTTCCACACTGCACCAAA) were designed using Primer‐BLAST [51] based on the information available in Gallus_gallus‐4.0 (MSMB3) and Gallus_gallus‐5.0 (MSMB1 and MSMB2) assemblies, and purchased from Eurogentec (Angers, France). Their gene specificity was validated by sequencing the corresponding PCR product (GENEWIZ, Paris, France). The cDNA (2.5 µL) was amplified using SYBR Green I Master Kit (Roche, Mannheim, Germany) and a Light Cycler 480 Apparatus (Roche Diagnostics, Meylan, France).

A melting curve program was carried out from 60 to 97 °C in 1 min for each individual sample. Each run included triplicates of control cDNA consisting of a pool of cDNA from all tissues. The control of pooled cDNA was diluted from 1 : 6.25 to 1 : 1 638 400, and relative arbitrary quantities were defined. A calibration curve was calculated using the threshold cycle (*C*_t_) values of the control cDNA samples to evaluate the relative amount of candidate genes. Chicken MSMB1, MSMB2, and MSMB3 gene expression levels were normalized by invariant housekeeping genes (TBP, EIF3I, and PPIA for female tissues and TBP, EIF3I, GUSB, B2M, SDHA, and GADPGH for male tissues) as follows. Levels of ‘TATA‐binding protein’ (TBP; NM_205103.1; Fw‐GCGTTTTGCTGCTGTTATTATGAG; Rev‐TCCTTGCTGCCAGTCTGGAC,), ‘eukaryotic translation initiation factor 3 subunit’ (EIF3I, NM_001164395.1; Fw‐GACATGTGCTCACTGGCTCT; Rev‐CACTGCTGAGCTGGTCTTCA), ‘peptidylprolyl isomerase A’ (PPIA, NM_001166326.1; Fw‐CGCTGACAAGGTGCCCATAA; Rev‐GTCACCACCCTGACACATGA) for female tissues, ‘glucuronidase beta’ (GUSB, NM_001039316.2; Fw‐TGTGATTGGGGAACTCATCTGG; Rev‐AAGTTCAGCATAGTACCCAGC), ‘beta‐2‐microglobulin’ (B2M, NM_001001750.3; Fw‐GATCCCGAGTTCTGAGCTGT; Rev‐GCTTGCTCTTTGCCGTCATAC), ‘succinate dehydrogenase complex flavoprotein subunit A’ (SDHA; NM_001277398.1; Fw‐AGATACGGGAAGGAAGGGGT; Rev‐ACCGTAGGCAAAACGGGAAT), and ‘glyceraldehyde‐3‐phosphate dehydrogenase’ (GADPH, NM_204305.1; Fw‐AGGCGAGATGGTGAAAGTCGGAGT; Rev‐TGCCCTTGAAGTGTCCGTGTGT) for male tissues were measured using SYBR Green reaction. The ratio value was calculated for each sample as MSMB1, MSMB2, or MSMB3 to geometric mean of TBP, EIF3I, and PPIA for female tissues and of TBP, EIF3I, GUSB, B2M, SDHA, and GADPGH for male tissues. Normalized quantities were calculated using the following formula: gene efficiency^(Ctcalibrator − Ctsample)/geometric average quantity of housekeeping genes.

### Mass spectrometry analysis of egg MSMB3 samples

Bottom‐up proteomic approach was performed to characterize sequence and potential post‐translational modifications. From 20 µg of purified MSMB3, reduction and cysteine alkylation were applied by successive incubations at final concentration of 5 mm dithiothreitol in 50 mm NH_4_HCO_3_ (30 min, 56 °C), and then at final concentration of 12.5 mm iodoacetamide in 50 mm NH_4_HCO_3_ (20 min, room temperature, in dark). In‐solution proteolytic digestion was carried out at 37 °C overnight using a final enzyme:substrate ratio of 1 : 100 with bovine trypsin (Sequencing Grade; Roche Diagnostics, Paris, France). The hydrolytic peptides were incubated with formic acid at a 1% final concentration. The resulting peptide mixture was concentrated and desalted with ziptips C18 (Millipore, Merck KGaA, Darmstadt, Germany) before analysis by on‐line nanoflow liquid chromatography–tandem mass spectrometry (nanoLC‐MS/MS).

All experiments were performed on a dual linear ion trap Fourier Transform Mass Spectrometer (FT‐MS) LTQ Orbitrap Velos Pro (Thermo Fisher Scientific, Bremen, Germany) coupled to an Ultimate® 3000 RSLC Ultra High‐Pressure Liquid Chromatographer (Thermo Fisher Scientific, Bremen, Germany), as previously described [31]. MS/MS ion searches were performed using Mascot search engine version 2.7.0.1 (Matrix Science, London, UK) via proteome discoverer 2.1 software (Thermo Fisher Scientific) using a homemade database that includes the chicken MSMB3 amino acid sequence. The selected parameters included trypsin as a protease with two allowed missed cleavages and carbamidomethylcysteine (+57 Da), methionine oxidation (+16 Da), amidation (−1), and acetylation (+42 Da) of amino‐terminal protein as variable modifications. The tolerance of the ions was set to 5 p.p.m. for parent and 0.8 Da for fragment ion matches.

Mascot results were subjected to scaffold software (v 4.11.1 Proteome Software; Portland, OR, USA) using the protein cluster analysis option (assemblage of proteins into clusters based on shared peptide evidence). Peptide identification and protein identification were validated by the peptide and protein prophet algorithms with specified probability greater than 99.9 and 99%, respectively.

Top‐down proteomic approach was performed from crude MSMB3 or after reduction and alkylation followed by desalting using Ziptip C4 (Millipore). On‐line microliquid chromatography–tandem mass spectrometry (µLC‐MS/MS) was carried out using the LTQ Orbitrap Velos Mass Spectrometer (Thermo Fisher Scientific) coupled to the Ultimate® 3000 RSLC System. Samples were loaded on a trap monolithic column (PS‐DVB PepSwift; 200 µm inner diameter × 5 mm long) and separated using a monolithic column (PS‐DVB PepSwift; 200 µm inner diameter × 5 cm long). Mobile phases consisted of (A) and (B) are the same as previously described for bottom‐up analysis. Proteins were preconcentrated for 10 min at 10 µL·min^−1^ with 4% solvent B. The gradient consisted of 4–10% B for 1 min, 10–75% B for 60 min, 75–99% B for 1 min, constant 99% B 20 min, and return to 4% B in 1 min. The flow rate was set to 1 µL·min^−1^. The eluate was sprayed using a SilicaTip emitter with 30 µm inner diameter and 360 µm outer diameter (New Objective, Woburn, MA, USA) into a Thermo Finnigan Nanospray Ion Source 1. Standard mass spectrometric conditions for all experiments were spray voltage 1.3 kV, no sheath, and auxiliary gas flow; heated capillary temperature, 250 °C; predictive automatic gain control enabled; and an S‐lens RF level of 70%. The LTQ Orbitrap Velos instrument was operated in positive mode, in the scan range of *m*/*z* 400–2000. The MS and MS/MS spectra were performed with a target resolution in the Orbitrap set to *r* = 100 000. Polydimethylcyclosiloxane (*m*/*z*, 445.1200025) ions were used for internal calibration.

Mass spectrometry/MS spectra of interest were manually extracted from Xcalibur and integrated to prosight pc software v 4.0 (Thermo Fisher, San Jose, CA, USA) for deconvolution using THRASH (signal/noise : 2) and submitted against the two MSMB3 sequences (with or without G terminal). Lists of monoisotopic masses of ions fragments were extracted and submitted to ProSight Lite tool with a mass tolerance at 10 ppm (at the fragment ion level) in order to note delta mass and scores.

Raw results from bottom‐up and top‐down mass spectrometry are available as Tables S2 and S3.

### NMR Spectroscopy of MSMB3

MSMB3 was dissolved in H_2_O : D_2_O (9 : 1 ratio) at a concentration of 300 or 50 μm in 100 mm Tris, pH 7.5, 50 mm NaCl.

Ubiquitin, lysozyme, myoglobin, and albumin were purchased from Sigma‐Aldrich and dissolved at a concentration of 100 µm in the same buffer as the MSMB3 protein.

All NMR experiments were performed on an Avance III HD Bruker 700 MHz Spectrometer equipped with a cryoprobe. NMR data were processed using Bruker's Topspin 3.2™ (Billerica, MA, USA) at 298 K. 1H and 2D 1H TOCSY (*T*_m_ = 80 ms) spectra were acquired on both samples of MSMB3 in order to assess whether the protein was highly structured.

DOSY experiments were acquired on all NMR samples using a standard Bruker sequence and diffusion protocol described in the NMR user manual. Calibration of the gradient strength was performed on a 99.9% D_2_O/0.1% GdCl3 sample. The physical observable that can be derived from the diffusion NMR experiment is the diffusion coefficient *D*, which is sensitive to the molecular mass of the molecular species. The empirically derived power law (Eqn 1) is probably the most powerful relation, which correlates the MW and the diffusion coefficient.

(1) $$D=K{\text{MW}}^{\alpha }$$

in which *K* is a molecule‐dependent constant and *α* is a coefficient that depends highly on the shape and type of the molecular species. Plotting the experimental diffusion coefficients of molecules or proteins, measured in the same buffer at the same temperature (Table S3), versus their known molecular mass in log–log scale, allowed us to obtain a calibration curve (Fig. S3C). Errors bars were estimated to be 7% for all measurements and reflect the experimental errors effectively measured on the diffusion coefficient of the Tris molecule present in all samples. A similar curve has been plotted with some diffusion coefficients found in the literature (Fig. S3D).

### X‐ray structure of MSMB3

MSMB3 was concentrated to 10 mg·mL^−1^ before initial sparse matrix crystallization screening using a Mosquito nanoliter pipetting robot (TTP Labtech Ltd., Melbourn, UK). The crystallization conditions were then manually refined using hanging drops to the final condition: 100 mm phosphate/citrate buffer pH 3.7, 100 mm Li_2_SO_4_, and 22% PEG1000. The crystals were grown at 20 °C for 2 weeks and then transferred into mother liquor supplemented with 25% ethylene glycol and flash‐frozen in liquid nitrogen. 100 K X‐ray data were collected at ESRF beamline ID29 and processed using XDS [52] and AIMLESS [53]. The 3D crystal structure of MSMB3 was determined at 2.14 Å resolution by molecular replacement with Phaser [54] of the Phenix suite [55] and using protein data bank (PDB) id 3ix0 (PSP94) as a search model. Atomic model was refined using phenix.refine and manually improved using COOT [56]. Data collection and refinement statistics are listed in Table 2. Molecular graphics images were produced using UCSF Chimera [57].

### MSMB1 and MSMB2 homology modeling

3D structure models of MSMB1 and MSMB2 were built from their primary protein sequence by homology modeling using I‐TASSER server [58]. All disulfide bonds were assigned as additional restraints to guide I‐TASSER modeling.

## Conflict of interest

The authors declare no conflict of interest.

## Author contributions

SR‐G and FC conceptualized the study; SR‐G, NG, FC, KL, and VL were involved in methodology; MC, MB, VL, and HM performed experiments and validated the data; SR‐G and FC wrote the original draft; SR‐G, FC, TM, NG, KL, VL, and BC wrote, reviewed, and edited the manuscript; SR‐G, VL, and BC acquired funding; SR‐G, FC, VL, KL, and BC provided resources; and SR‐G and FC supervised the data.

## Supporting information


**Fig. S1**. Multicharged‐ion spectra for chicken MSMB3 proteoform lacking a G residue.
**Fig. S2**. Multicharged‐ion spectra for native chicken MSMB3.
**Fig. S3**. Sequences and fragmentation patterns of the chicken egg purified MSMB3 proteoforms.
**Fig. S4**. Oligomeric state of MSMB3 in solution.
**Fig. S5**. Residue‐residue interactions across dimer interfaces.
**Fig. S6**. Interactions of sulfate ions with MSMB3 homodimer.

**Table S1**. Values of diffusion coefficient for various proteins in water.

**Table S2**. Bottom‐up data.

**Table S3**. Top‐down data.

## Data Availability

Atomic coordinates and structure factors for MSMB3 crystal have been deposited in the PDB with the Accession Number 6rwc.

## References

[1] Frankenberg S , Fenelon J , Dopheide B , Shaw G and Renfree MB (2011) A novel MSMB‐related microprotein in the postovulatory egg coats of marsupials. BMC Evol Biol 11, 373. 22208949 10.1186/1471-2148-11-373 PMC3268785

[2] Aoki N , Sakiyama A , Deshimaru M and Terada S (2007) Identification of novel serum proteins in a Japanese viper: homologs of mammalian PSP94. Biochem Biophys Res Commun 359, 330–334. 17543280 10.1016/j.bbrc.2007.05.091

[3] Valtonen‐Andre C and Lundwall A (2008) The cotton‐top tamarin (Saguinus oedipus) has five beta‐microseminoprotein genes, two of which are pseudogenes. DNA Cell Biol 27, 45–54. 18020964 10.1089/dna.2007.0641

[4] Makinen M , Valtonen‐Andre C and Lundwall A (1999) New world, but not Old World, monkeys carry several genes encoding beta‐microseminoprotein. Eur J Biochem 264, 407–414. 10491085 10.1046/j.1432-1327.1999.00614.x

[5] Lundwall A , Larne O , Nayudu PL , Ceder Y and Valtonen‐Andre C (2009) Rapidly evolving marmoset MSMB genes are differently expressed in the male genital tract. Reprod Biol Endocrinol 7, 96. 19737427 10.1186/1477-7827-7-96 PMC2746217

[6] Valtonen‐Andre C , Bjartell A , Hellsten R , Lilja H , Harkonen P and Lundwall A (2007) A highly conserved protein secreted by the prostate cancer cell line PC‐3 is expressed in benign and malignant prostate tissue. Biol Chem 388, 289–295. 17338636 10.1515/BC.2007.032

[7] Lazure C , Villemure M , Gauthier D , Naude RJ and Mbikay M (2001) Characterization of ostrich (Struthio camelus) beta‐microseminoprotein (MSP): identification of homologous sequences in EST databases and analysis of their evolution during speciation. Protein Sci 10, 2207–2218. 11604528 10.1110/ps.06501 PMC2374068

[8] Wang I , Yu TA , Wu SH , Chang WC and Chen C (2003) Disulfide pairings and secondary structure of porcine beta‐microseminoprotein. FEBS Let 541, 80–84. 12706823 10.1016/s0014-5793(03)00308-9

[9] Ghasriani H , Teilum K , Johnsson Y , Fernlund P and Drakenberg T (2006) Solution structures of human and porcine beta‐microseminoprotein. J Mol Biol 362, 502–515. 16930619 10.1016/j.jmb.2006.07.029

[10] Shioi N , Tadokoro T , Shioi S , Okabe Y , Matsubara H , Kita S , Ose T , Kuroki K , Terada S and Maenaka K (2019) Crystal structure of the complex between venom toxin and serum inhibitor from Viperidae snake. J Biol Chem 294, 1250–1256. 30504218 10.1074/jbc.RA118.006840 PMC6349104

[11] Kumar V , Roske Y , Singh N , Heinemann U , Singh TP and Yadav S (2009) Purification and preliminary X‐ray crystallographic studies of beta‐microseminoprotein from human seminal plasma. Acta Crystallogr Sect F Struct Biol Cryst Commun 65, 518–521. 10.1107/S1744309109013670 PMC2675600 19407392

[12] Kumar A , Jagtap DD , Mahale SD and Kumar M (2010) Crystal structure of prostate secretory protein PSP94 shows an edge‐to‐edge association of two monomers to form a homodimer. J Mol Biol 397, 947–956. 20184897 10.1016/j.jmb.2010.02.035

[13] Akiyama K , Yoshioka Y , Schmid K , Offner GD , Troxler RF , Tsuda R and Hara M (1985) The amino acid sequence of human beta‐microseminoprotein. Biochim Biophy Acta 829, 288–294. 10.1016/0167-4838(85)90200-6 3995056

[14] Hara M and Kimura H (1989) Two prostate‐specific antigens, gamma‐seminoprotein and beta‐microseminoprotein. J Lab Clin Med 113, 541–548. 2654306

[15] Anklesaria JH , Mhatre DR and Mahale SD (2018) Structural and molecular biology of PSP94: Its significance in prostate pathophysiology. Front Biosci (Landmark edition) 23, 535–562. 10.2741/4604 28930560

[16] Weiber H , Andersson C , Murne A , Rannevik G , Lindstrom C , Lilja H and Fernlund P (1990) Beta microseminoprotein is not a prostate‐specific protein. Its identification in mucous glands and secretions. Am J Pathol 137, 593–603. 2205099 PMC1877516

[17] Udby L , Lundwall A , Johnsen AH , Fernlund P , Valtonen‐Andre C , Blom AM , Lilja H , Borregaard N , Kjeldsen L and Bjartell A (2005) beta‐Microseminoprotein binds CRISP‐3 in human seminal plasma. Biochem Biophys Res Commun 333, 555–561. 15950934 10.1016/j.bbrc.2005.05.139 PMC1939934

[18] Moreau R , Frank PG , Perreault C , Marcel YL and Manjunath P (1999) Seminal plasma choline phospholipid‐binding proteins stimulate cellular cholesterol and phospholipid efflux. Biochim Biophys Acta 1438, 38–46. 10216278 10.1016/s1388-1981(99)00032-3

[19] Manaskova‐Postlerova P , Davidova N , Sulc M , Philimonenko A , Hozak P and Jonakova V (2011) Reproductive tissue expression and sperm localization of porcine beta‐microseminoprotein. Cell Tissue Res 344, 341–353. 21384183 10.1007/s00441-011-1149-y

[20] Lane M , Therien I , Moreau R and Manjunath P (1999) Heparin and high‐density lipoprotein mediate bovine sperm capacitation by different mechanisms. Biol Reprod 60, 169–175. 9858502 10.1095/biolreprod60.1.169

[21] Hansson K , Kjellberg M and Fernlund P (2009) Cysteine‐rich secretory proteins in snake venoms form high affinity complexes with human and porcine beta‐microseminoproteins. Toxicon 54, 128–137. 19341830 10.1016/j.toxicon.2009.03.023

[22] Desnoyers L and Manjunath P (1992) Major proteins of bovine seminal plasma exhibit novel interactions with phospholipid. J Biol Chem 267, 10149–10155. 1577785

[23] Anklesaria JH , Pandya RR , Pathak BR and Mahale SD (2016) Purification and characterization of CRISP‐3 from human seminal plasma and its real‐time binding kinetics with PSP94. J Chromatogr B Analyt Technol Biomed Life Sci 1039, 59–65. 10.1016/j.jchromb.2016.10.032 27825912

[24] Anahi Franchi N , Avendano C , Molina RI , Tissera AD , Maldonado CA , Oehninger S and Coronel CE (2008) beta‐Microseminoprotein in human spermatozoa and its potential role in male fertility. Reproduction (Cambridge, England) 136, 157–166. 10.1530/REP-08-0032 18469041

[25] Baijal‐Gupta M , Clarke MW , Finkelman MA , McLachlin CM and Han VK (2000) Prostatic secretory protein (PSP94) expression in human female reproductive tissues, breast and in endometrial cancer cell lines. J Endocrinol 165, 425–433. 10810306 10.1677/joe.0.1650425

[26] Guyot N , Labas V , Harichaux G , Chesse M , Poirier JC , Nys Y and Rehault‐Godbert S (2016) Proteomic analysis of egg white heparin‐binding proteins: towards the identification of natural antibacterial molecules. Sci Rep 6, 27974. 27294500 10.1038/srep27974 PMC4904793

[27] Edstrom Hagerwall AM , Rydengard V , Fernlund P , Morgelin M , Baumgarten M , Cole AM , Malmsten M , Kragelund BB and Sorensen OE (2012) beta‐Microseminoprotein endows post coital seminal plasma with potent candidacidal activity by a calcium‐ and pH‐dependent mechanism. PLoS Pathog 8, e1002625. 22496651 10.1371/journal.ppat.1002625 PMC3320615

[28] Yang WC , Kwok SC , Leshin S , Bollo E and Li WI (1998) Purified porcine seminal plasma protein enhances *in vitro* immune activities of porcine peripheral lymphocytes. Biol Reprod 59, 202–207. 9675013 10.1095/biolreprod59.1.202

[29] Derwenskus KH , Sprinzl M and Scheit KH (1989) Inhibition of cell proliferation by basic proteins from bull seminal plasma. Biol Chem Hoppe Seyler 370, 284–292. 2474303

[30] Mann K , Macek B and Olsen JV (2006) Proteomic analysis of the acid‐soluble organic matrix of the chicken calcified eggshell layer. Proteomics 6, 3801–3810. 16767793 10.1002/pmic.200600120

[31] Labas V , Grasseau I , Cahier K , Gargaros A , Harichaux G , Teixeira‐Gomes AP , Alves S , Bourin M , Gerard N and Blesbois E (2015) Qualitative and quantitative peptidomic and proteomic approaches to phenotyping chicken semen. J Proteomics 112, 313–335. 25086240 10.1016/j.jprot.2014.07.024

[32] Da Silva M , Dombre C , Brionne A , Monget P , Chesse M , De Pauw M , Mills M , Combes‐Soia L , Labas V , Guyot N *et al*. (2019) The unique features of proteins depicting the chicken amniotic fluid. Mol Cell Proteomics 18, S174–S190. 29444982 10.1074/mcp.RA117.000459 PMC6427230

[33] Karandikar A and Ghaskadbi S (2003) beta‐Microseminoprotein‐related molecules may participate in formation of the mesoderm in the chick embryo. Dev Growth Differ 45, 309–319. 12950272 10.1046/j.1440-169x.2003.00698.x

[34] Wang Y , Zhang S , Liu Z , Li H and Wang L (2005) Identification and expression of amphioxus beta‐microseminoprotein (MSP)‐like gene encoding an ancient and rapidly evolving protein in chordates. Comparative biochemistry and physiology Part B. Biochem Mol Biol 142, 251–257. 10.1016/j.cbpb.2005.07.014 16150623

[35] Wang C , Qi X , Zhou X , Sun J , Cai D , Lu G , Chen X , Jiang Z , Yao YG , Chan WY *et al*. (2020) RNA‐Seq analysis on ets1 mutant embryos of Xenopus tropicalis identifies microseminoprotein beta gene 3 as an essential regulator of neural crest migration. FASEB J 34, 12726–12738. 32713114 10.1096/fj.202000603R

[36] Speir ML , Zweig AS , Rosenbloom KR , Raney BJ , Paten B , Nejad P , Lee BT , Learned K , Karolchik D , Hinrichs AS *et al*. (2016) The UCSC Genome Browser database: 2016 update. Nucleic Acids Res 44, D717–D725. 26590259 10.1093/nar/gkv1275 PMC4702902

[37] Altschul SF , Wootton JC , Gertz EM , Agarwala R , Morgulis A , Schaffer AA and Yu YK (2005) Protein database searches using compositionally adjusted substitution matrices. FEBS J 272, 5101–5109. 16218944 10.1111/j.1742-4658.2005.04945.x PMC1343503

[38] Fagerberg L , Hallstrom BM , Oksvold P , Kampf C , Djureinovic D , Odeberg J , Habuka M , Tahmasebpoor S , Danielsson A , Edlund K *et al*. (2014) Analysis of the human tissue‐specific expression by genome‐wide integration of transcriptomics and antibody‐based proteomics. Mol Cell Proteomics 13, 397–406. 10.1074/mcp.M113.035600 PMC3916642 24309898

[39] Marie P , Labas V , Brionne A , Harichaux G , Hennequet‐Antier C , Nys Y and Gautron J (2015) Quantitative proteomics and bioinformatic analysis provide new insight into protein function during avian eggshell biomineralization. J Proteomics 113, 178–193. 25284052 10.1016/j.jprot.2014.09.024

[40] Ahmed TAE , Suso H‐P and Hincke MT (2019) Experimental datasets on processed eggshell membrane powder for wound healing. Data Brief 26, 104457. 31667229 10.1016/j.dib.2019.104457 PMC6811977

[41] Rose‐Martel M , Smiley S and Hincke MT (2015) Novel identification of matrix proteins involved in calcitic biomineralization. J Proteomics 116, 81–96. 25585129 10.1016/j.jprot.2015.01.002

[42] Rehault‐Godbert S , Herve‐Grepinet V , Gautron J , Cabau C , Nys Y and Hincke M (2011) Molecules involved in chemical defence of the chicken egg. In Improving the Safety and Quality of Eggs and Egg Products, Vol 1: Egg Chemistry, Production and Consumption ( Nys Y , Bain M and VanImmerseel F , eds), pp. 183–208.Woodhead Publ Ltd, Cambridge.

[43] Saxena I and Tayyab S (1997) Protein proteinase inhibitors from avian egg whites. Cell Mol Life Sci 53, 13–23. 9117993 10.1007/PL00000575 PMC11147361

[44] Szecsi PB and Lilja H (1993) Gastricsin‐mediated proteolytic degradation of human seminal fluid proteins at pH levels found in the human vagina. J Androl 14, 351–358. 7507100

[45] Mann K and Mann M (2011) In‐depth analysis of the chicken egg white proteome using an LTQ Orbitrap Velos. Proteome Sci 9, 7. 21299891 10.1186/1477-5956-9-7 PMC3041730

[46] Mori H , Kamada M , Maegawa M , Yamamoto S , Aono T , Futaki S , Yano M , Kido H and Koide SS (1998) Enzymatic activation of immunoglobulin binding factor in female reproductive tract. Biochem Biophys Res Commun 246, 409–413. 9610373 10.1006/bbrc.1998.8633

[47] Kamada M , Liang Z and Koide SS (1991) Identification of IgG and Fc‐binding proteins in human seminal plasma and sperm. Arch Androl 27, 1–7. 1837706 10.3109/01485019108987645

[48] Liang ZG , Kamada M and Koide SS (1993) Binding of a specific subclass of immunoglobulins by a human seminal plasma component. Andrologia 25, 279–282. 8250291 10.1111/j.1439-0272.1993.tb02725.x

[49] Haas HJ and Spratt NT Jr (1976) Contributions to an analysis of the avian vitelline membrane's potential to promote outgrowth of the yolk sac‐serosal membrane. Anat Rec 184, 227–231. 942818 10.1002/ar.1091840208

[50] Thelie A , Rehault‐Godbert S , Poirier JC , Govoroun M , Fouchecourt S and Blesbois E (2019) The seminal acrosin‐inhibitor ClTI1/SPINK2 is a fertility‐associated marker in the chicken. Mol Reprod Dev 86, 762–775. 31033055 10.1002/mrd.23153 PMC6767445

[51] Ye J , Coulouris G , Zaretskaya I , Cutcutache I , Rozen S and Madden TL (2012) Primer‐BLAST: a tool to design target‐specific primers for polymerase chain reaction. BMC Bioinformatics 13, 11. 22708584 10.1186/1471-2105-13-134 PMC3412702

[52] Kabsch W (2010) XDS. Acta Crystallogr D Biol Crystallogr 66, 125–132. 20124692 10.1107/S0907444909047337 PMC2815665

[53] Evans PR and Murshudov GN (2013) How good are my data and what is the resolution? Acta Crystallogr D Biol Crystallogr 69, 1204–1214. 23793146 10.1107/S0907444913000061 PMC3689523

[54] McCoy AJ (2007) Solving structures of protein complexes by molecular replacement with Phaser. Acta Crystallogr D Biol Crystallogr 63, 32–41. 17164524 10.1107/S0907444906045975 PMC2483468

[55] Adams PD , Afonine PV , Bunkoczi G , Chen VB , Davis IW , Echols N , Headd JJ , Hung LW , Kapral GJ , Grosse‐Kunstleve RW *et al*. (2010) PHENIX: a comprehensive Python‐based system for macromolecular structure solution. Acta Crystallogr D Biol Crystallogr 66, 213–221. 20124702 10.1107/S0907444909052925 PMC2815670

[56] Emsley P and Cowtan K (2004) Coot: model‐building tools for molecular graphics. Acta Crystallogr D Biol Crystallogr 60, 2126–2132. 15572765 10.1107/S0907444904019158

[57] Pettersen EF , Goddard TD , Huang CC , Couch GS , Greenblatt DM , Meng EC and Ferrin TE (2004) UCSF Chimera–a visualization system for exploratory research and analysis. J Comput Chem 25, 1605–1612. 15264254 10.1002/jcc.20084

[58] Roy A , Kucukural A and Zhang Y (2010) I‐TASSER: a unified platform for automated protein structure and function prediction. Nat Protoc 5, 725–738. 20360767 10.1038/nprot.2010.5 PMC2849174

[59] Baker NA , Sept D , Joseph S , Holst MJ and McCammon JA (2001) Electrostatics of nanosystems: application to microtubules and the ribosome. Proc Natl Acad Sci USA 98, 10037–10041. 11517324 10.1073/pnas.181342398 PMC56910

[60] Dolinsky TJ , Nielsen JE , McCammon JA and Baker NA (2004) PDB2PQR: an automated pipeline for the setup of Poisson‐Boltzmann electrostatics calculations. Nucleic Acids Res 32, W665–W667. 15215472 10.1093/nar/gkh381 PMC441519

