# Supplementary material for: Three‐dimensional structures of avian beta‐microseminoproteins: insight from the chicken egg‐specific beta‐microseminoprotein 3 paralog
Source: FEBS Open Bio. 2021 May 24;11(6):1739–56. doi: 10.1002/2211-5463.13166 (PMC8167871; doi:10.1002/2211-5463.13166)
Supplement: Supplementary file 1 — Fig. S1. Multicharged‐ion spectra for chicken MSMB3 proteoform lacking a G residue. Fig. S2. Multicharged‐ion spectra for native chicken MSMB3. Fig. S3. Sequences and fragmentation patterns of the chicken egg purified MSMB3 proteoforms. Fig. S4. Oligomeric state of MSMB3 in solution. Fig. S5. Residue‐residue interactions across dimer interfaces. Fig. S6. Interactions of sulfate ions with MSMB3 homodimer. [file FEB4-11-1739-s001.docx]

**Supporting information**

**Three-dimensional structures of avian beta-microseminoproteins: insight from the chicken egg-specific beta-microseminoprotein 3 paralog**

Franck Coste, Thierry Moreau, Valérie Labas, Magali Chessé, Mégane Bregeon, Hervé Meudal, Karine Loth, Bertrand Castaing, Nicolas Guyot, Sophie Réhault-Godbert

List of material included:

Figure S1. Multicharged-ion spectra for chicken MSMB3 proteoform lacking a G residue.

Figure S2. Multicharged-ion spectra for native chicken MSMB3.

Figure S3. Sequences and fragmentation patterns of the chicken egg purified MSMB3 proteoforms.

Figure S4. Oligomeric state of MSMB3 in solution.

Figure S5. Residue-residue interactions across dimer interfaces.

Figure S6. Interactions of sulfate ions with MSMB3 homodimer.


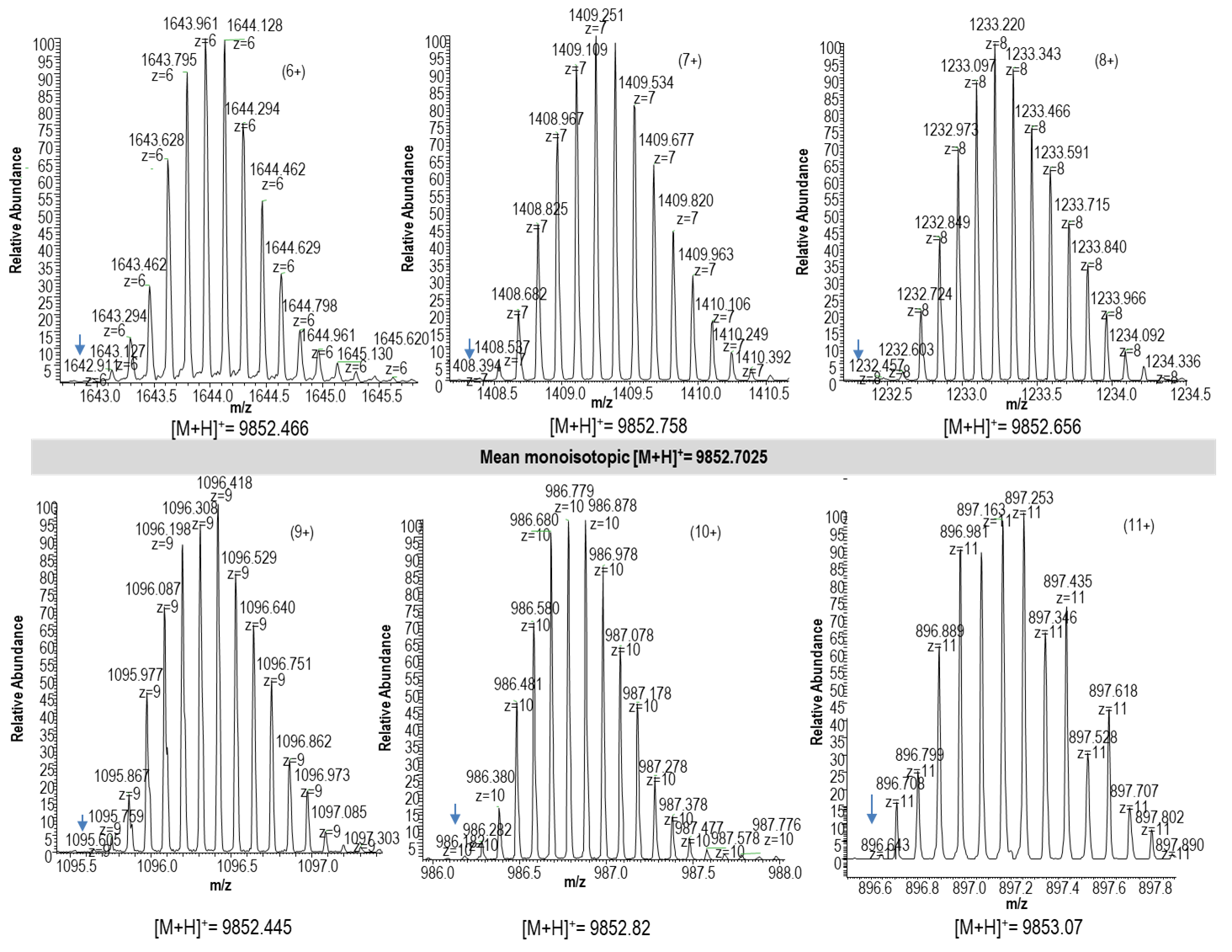


**Figure S1.** Multicharged-ion spectra for chicken MSMB3 proteoform lacking a G residue. The monoisotopic molecular mass of the chicken MSMB3 variant was observed at m/z 1642.911 (+6), 1408.394 (+7), 1232.457 (+8), 1095.605 (+9), 986.182 (+10), and 896.643 (+11). These monoisotopic masses allowed to calculate a mean monoisotopic [M+H]+= 9852.7025 for native MSMB3.


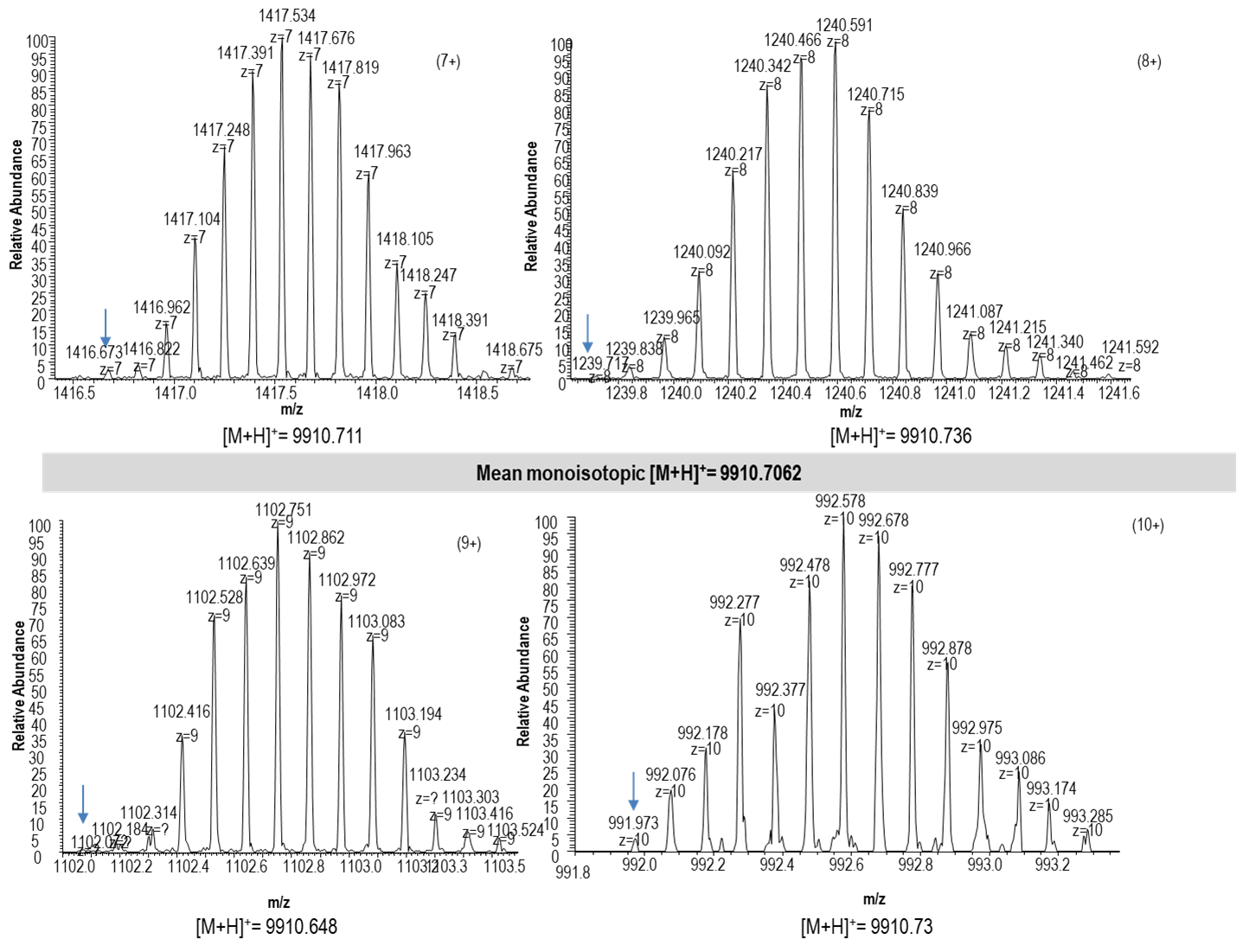


**Figure S2.** Multicharged-ion spectra for native chicken MSMB3. The monoisotopic molecular mass of the native chicken MSMB3 was observed at m/z 991.973, 1102.072, 1239.717 and 1416.673 for the (+7), (+8), (+9), (+10) charge states, respectively (black arrow). These monoisotopic masses allowed to calculate a mean monoisotopic [M+H]+= 9910.7062 for native MSMB3.


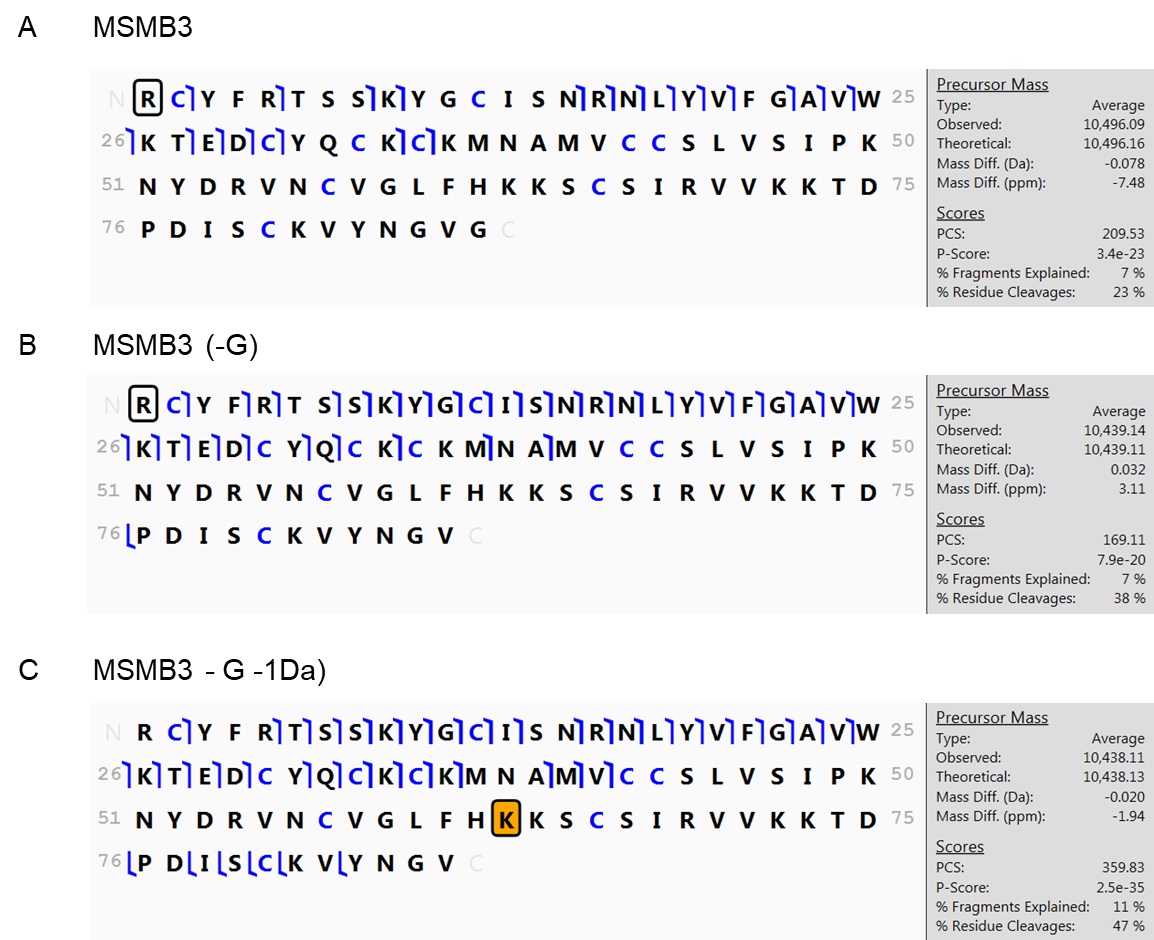


**Figure S3**. Sequences and fragmentation patterns of the chicken egg purified MSMB3 proteoforms. MS/MS information results from the fragmentation of one or several multicharged precursors : (12+) for MSMB3, (10+, 14+, 15) producing combined coverage sequence for MSMB3 (-G) and (8+, 9+, 11+, 13+) for MSMB3 (-G) – 1 Da.

**
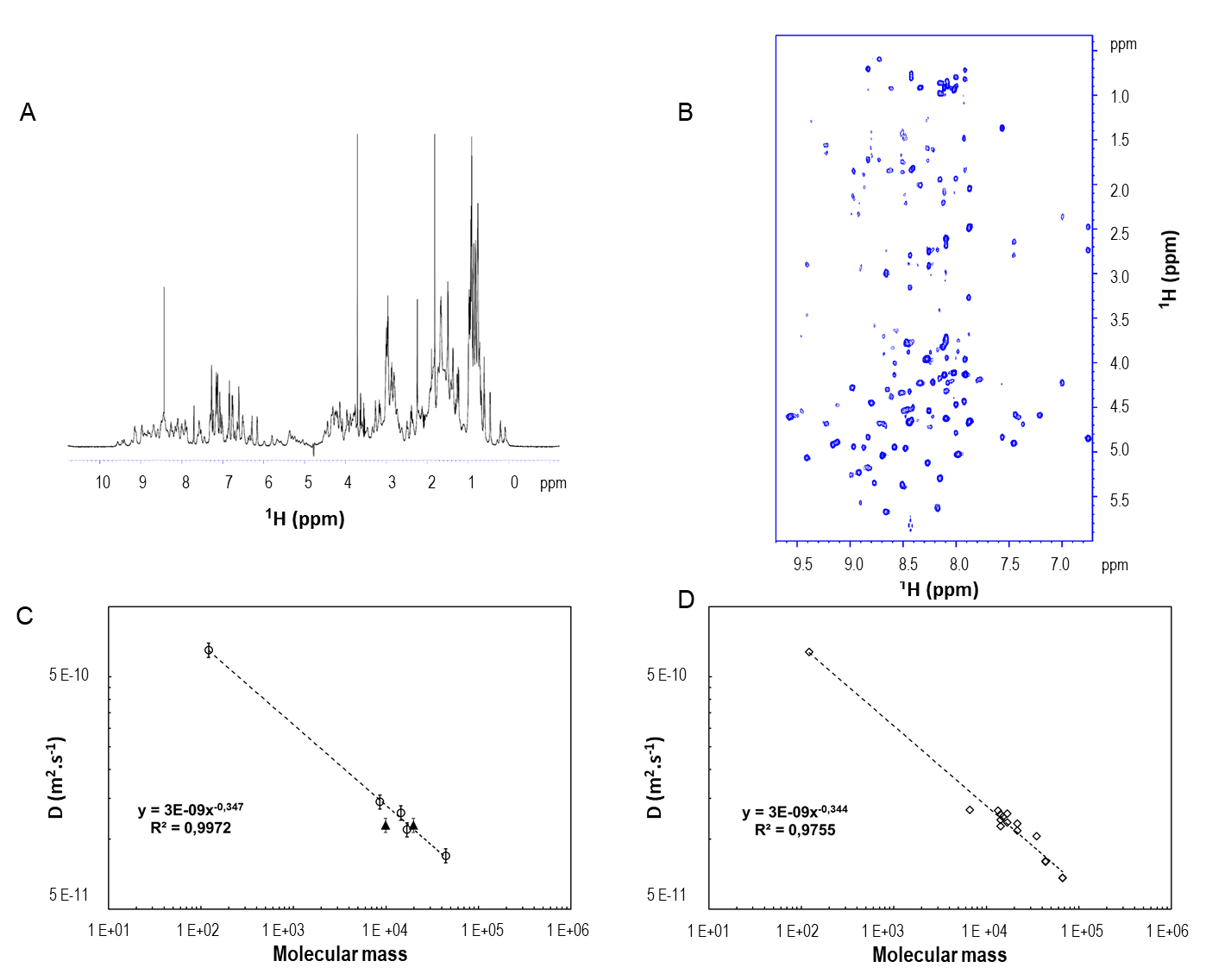
**

**Figure S4. Oligomeric state of MSMB3 in solution.** A. ^1^H NMR spectrum of the MSMB3 protein. B. Amide region of the 2D ^1^H TOCSY spectrum of the MSMB3 protein. C. Calibration curve to determine the oligomeric state of the MSMB3 protein in solution. Circles represent the diffusion coefficients of molecules of known molecular mass and oligomeric state. The data points were fitted by a power law. The equation and the R^2^ value are indicated on the graph. Triangles represent the diffusion coefficient of the MSMB3 protein depending on the monomer or dimer status of the protein. Error bars were estimated to be 7%. D. Calibration curve derived from values of diffusion coefficients found in the literature. The data points were fitted by a power law. The equation and the R^2^ value are indicated on the graph.

**
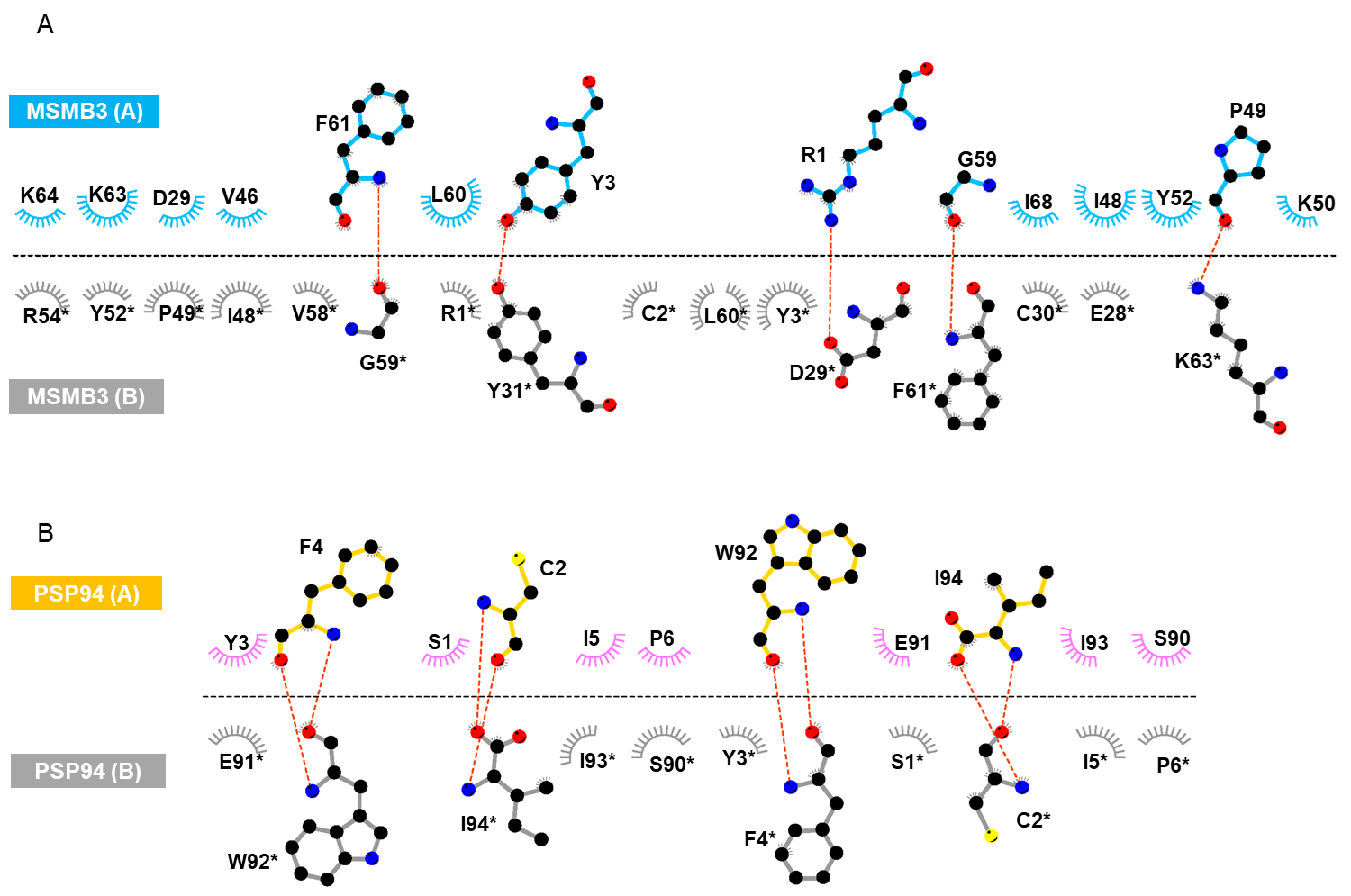
**

**Figure S5. Residue-residue interactions across dimer interfaces**. A. Ligplot+ representation of residue-residue interactions across MSMB3 homodimer (protein chain A in cyan and chain B in gray). Interactions plotted include hydrogen bonds (orange dashed line) and residues making non-bonded contacts (spoked arcs). Default parameters of the DIMPLOT module of Ligplot+ software were used for calculations (1). B. same as A. but for PSP94 homodimer (yellow/gray).

1. Laskowski, R. A., and Swindells, M. B. (2011) LigPlot+: multiple ligand-protein interaction diagrams for drug discovery. Journal of chemical information and modeling 51, 2778-2786

**
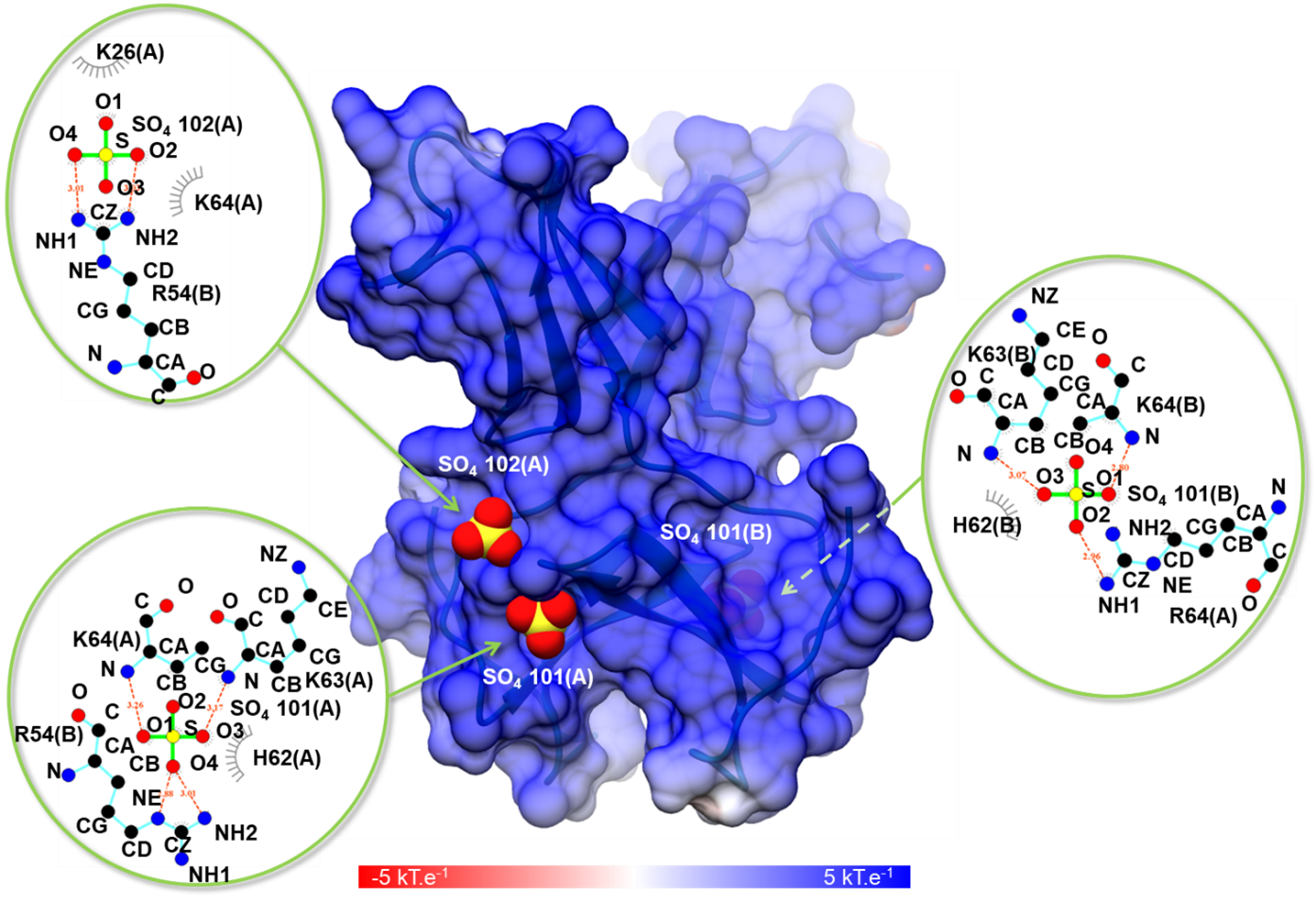
**

**Figure S6.** **Interactions of sulfate ions with MSMB3 homodimer.** MSMB3 is represented as a semi-transparent molecular surface colored by electrostatic potential; sulfate ions are represented as CPK models (yellow for sulfur and red for oxygen atoms). Green boxes show close-up views of ion-residue interactions calculated using Ligplot+ (Laskowski and Swindells, 2011). Interactions plotted include hydrogen bonds (orange dashed line) and residues making non-bonded contacts (spoked arcs).
