# Supplementary material for: Three‐dimensional structures of avian beta‐microseminoproteins: insight from the chicken egg‐specific beta‐microseminoprotein 3 paralog
Source: FEBS Open Bio. 2021 May 24;11(6):1739–56. doi: 10.1002/2211-5463.13166 (PMC8167871; doi:10.1002/2211-5463.13166)
Supplement: Supplementary file 2 — Table S1. Values of diffusion coefficient for various proteins in water. [file FEB4-11-1739-s002.docx]

**Table S1**: **Values of diffusion coefficient for various proteins in water** (all values corrected to 25°C using *Dη*/T = constant when necessary), adapted from (1). * Measurements corresponding with the DOSY experiments of this study (Errors were estimated to be 7%, see Material and Methods section).

| **Protein** | **MW (kDa)** | **D (x 10^-10^ m^2^.s^-1^)** |
| --- | --- | --- |
| Tris | 0.121 | 6.31 |
| IGF-1 (2) | 7.6 | 1.50 |
| Ubiquitin* | 8.6 | 1.45 |
| MSMB3* | 9.9 / 18.8 | 1.29 |
| cytochrome c (3) | 13.4 | 1.33 |
| Lysozyme (4) | 14.3 | 1.28 |
| Lysozyme* | 14.5 | 1.30 |
| α-Lactalbumin (5) | 14.2 | 1.21 |
| Lactalbumin (3) | 14.2 | 1.14 |
| Trypsin (6) | 15.1 | 1.25 |
| Myoglobin* | 16.9 | 1.10 |
| Myoglobin (4) | 16.9 | 1.18 |
| Myoglobin (6) | 16.9 | 1.29 |
| α-Chymotrypsin (4) | 21.6 | 1.17 |
| Chymotrypsinogen (4) | 21.6 | 1.09 |
| Pepsin (7) | 35.0 | 1.03 |
| Ovalbumin (3,4,6) | 43.5 | 0.78 – 0.83 |
| Albumin* | 44.3 | 0.85 |
| BSA (3,4,8) | 66.5 | 0.64-0.72 |

1. Nauman, J. V., Campbell, P. G., Lanni, F., and Anderson, J. L. (2007) Diffusion of insulin-like growth factor-I and ribonuclease through fibrin gels. *Biophysical journal* **92**, 4444-4450

2. Schneiderman, R., Snir, E., Popper, O., Hiss, J., Stein, H., and Maroudas, A. (1995) Insulin-like growth factor-I and its complexes in normal human articular cartilage: studies of partition and diffusion. *Archives of biochemistry and biophysics* **324**, 159-172

3. Walters, R. R., Graham, J. F., Moore, R. M., and Anderson, D. J. (1984) Protein diffusion coefficient measurements by laminar flow analysis: method and applications. *Analytical biochemistry* **140**, 190-195

4. Tyn, M. T., and Gusek, T. W. (1990) Prediction of diffusion coefficients of proteins. *Biotechnology and bioengineering* **35**, 327-338

5. Saltzman, W. M., Radomsky, M. L., Whaley, K. J., and Cone, R. A. (1994) Antibody diffusion in human cervical mucus. *Biophysical journal* **66**, 508-515

6. Sober, H. A. (1970) *Handbook of Biochemistry*, CRC Press, Cleverland, OH, U.S.A.

7. Liu, M. K., Li, P., and Giddings, J. C. (1993) Rapid protein separation and diffusion coefficient measurement by frit inlet flow field-flow fractionation. *Protein science : a publication of the Protein Society* **2**, 1520-1531

8. Cantor, C. R., and Schimmel, P. R. (1980). in *Biophysical chemistry: Part II ‘Techniques for the study of biological structure and function’*, WH Freeman and Co, Oxford, U.K.
